# Modeling functional connectivity with learning and memory in a mouse model of Alzheimer's disease

**DOI:** 10.3389/fnimg.2025.1558759

**Published:** 2025-04-25

**Authors:** Lindsay Fadel, Elizabeth Hipskind, Steen E. Pedersen, Jonathan Romero, Caitlyn Ortiz, Eric Shin, Md Abul Hassan Samee, Robia G. Pautler

**Affiliations:** ^1^Department of Neuroscience, Baylor College of Medicine, Houston, TX, United States; ^2^Department of Integrative Physiology, Baylor College of Medicine, Houston, TX, United States; ^3^Small Animal Imaging Facility, Texas Children's Hospital, Houston, TX, United States

**Keywords:** Alzheimer's disease, rs-fMRI, functional connectivity, modeling, behavior, mouse model

## Abstract

**Introduction:**

Functional connectivity (FC) is a metric of how different brain regions interact with each other. Although there have been some studies correlating learning and memory with FC, there have not yet been, to date, studies that use machine learning (ML) to explain how FC changes can be used to explain behavior not only in healthy mice, but also in mouse models of Alzheimer's Disease (AD). Here, we investigated changes in FC and their relationship to learning and memory in a mouse model of AD across disease progression.

**Methods:**

We assessed the APP/PS1 mouse model of AD and wild-type controls at 3-, 6-, and 10-months of age. Using resting state functional magnetic resonance imaging (rs-fMRI) in awake, unanesthetized mice, we assessed FC between 30 brain regions. ML models were then used to define interactions between neuroimaging readouts with learning and memory performance.

**Results:**

In the APP/PS1 mice, we identified a pattern of hyperconnectivity across all three time points, with 47 hyperconnected regions at 3 months, 46 at 6 months, and 84 at 10 months. Notably, FC changes were also observed in the Default Mode Network, exhibiting a loss of hyperconnectivity over time. Modeling revealed functional connections that support learning and memory performance differ between the 6- and 10-month groups.

**Discussion:**

These ML models show potential for early disease detection by identifying connectivity patterns associated with cognitive decline. Additionally, ML may provide a means to begin to understand how FC translates into learning and memory performance.

## 1 Introduction

Alzheimer's disease (AD) is a progressive neurodegenerative disorder impairing cognitive function and memory (Scheltens et al., 2021). A conclusive AD diagnosis requires postmortem examination to confirm the presence of Aβ plaques, tau tangles, and atrophy (McLean et al., 1997). In living patients, a probable diagnosis involves cognitive assessments, plasma Aβ measurements, and PET imaging to assess Aβ plaque and tau tangle deposition (Coupé et al., 2019; Cullen et al., 2021; Manera et al., 2023). Despite these indirect tests, there is no definitive diagnostic available for early and accurate AD diagnosis (Burnham et al., 2024). However, advancements in neuroimaging techniques that can detect deficits prior to significant accumulation of pathological features and cognitive decline would aid early detection, diagnosis, and potential therapeutic intervention of AD (Gauthier, 2005; Jagust, 2018; Leuzy et al., 2019; Olivari et al., 2020).

Magnetic resonance imaging (MRI) has emerged as a promising diagnostic technique for neurodegenerative disease, offering the ability to non-invasively measure both anatomical and functional connectivity (FC). Resting state functional MRI (rs-fMRI) is a type of functional MRI that is task independent and uses blood oxygen level detection (BOLD) to map FC, the correlation of activity that occurs between spatially separated brain regions.

In both AD patients and mouse models, rs-fMRI has revealed deficits in FC in brain regions that are involved in memory functions (Wang et al., 2013; Xu et al., 2024). Understanding patterns of resting-state connectivity provides insights into brain function, cognitive processes, and potential mechanisms underlying various neurological disorders (Horin et al., 2021; Weingarten and Strauman, 2015). Alterations in FC have been shown to emerge prior to cognitive deficits or significant Aβ plaque or tau tangle accumulation, underscoring their potential as early markers of AD progression (Chen et al., 2022; Kesler et al., 2018; Morrissey et al., 2022).

Several specific FC changes have been identified through rsfMRI studies in individuals with AD. One prominent alteration involves the default mode network (DMN), a network of brain regions associated with introspection, memory retrieval, and self-referential processing (Andrews-Hanna et al., 2014; Buckner and DiNicola, 2019). This network is highly conserved across several mammalian species including humans, non-human primates, rats, and mice, suggesting its fundamental role in cognitive processes and resting state functions (Pagani et al., 2023). Changes in DMN connectivity have been observed in AD patients, correlating with cognitive decline and disease progression (Ibrahim et al., 2021; Petrella et al., 2011). Neuropathological studies have revealed the accumulation of Aβ plaques and tau tangles in key DMN regions, contributing to neuronal dysfunction and cognitive decline (Giorgio et al., 2024; Palmqvist et al., 2017).

Changes in rsFC have been linked to the underlying histopathological hallmarks of AD, including the accumulation of Aβ plaques and tau tangles. Recent human PET imaging studies also reveal a correlation between tau tangle accumulation and functional hypoconnectivity in the brain, with Aβ plaque deposition being associated with functional hyperconnectivity (Schultz et al., 2017; Sepulcre et al., 2017; Sintini et al., 2021; Wales and Leung, 2021). Since PET tracers bind to protein aggregates, PET imaging studies are unable to assess effects of hyperphosphorylated tau or Aβ before aggregation, which limits its utility as an early diagnostic method (Mathis et al., 2017). Early diagnosis, prior to the aggregation of these proteins, is crucial for developing effective interventions and potentially slowing disease progression.

**Figure 1:**
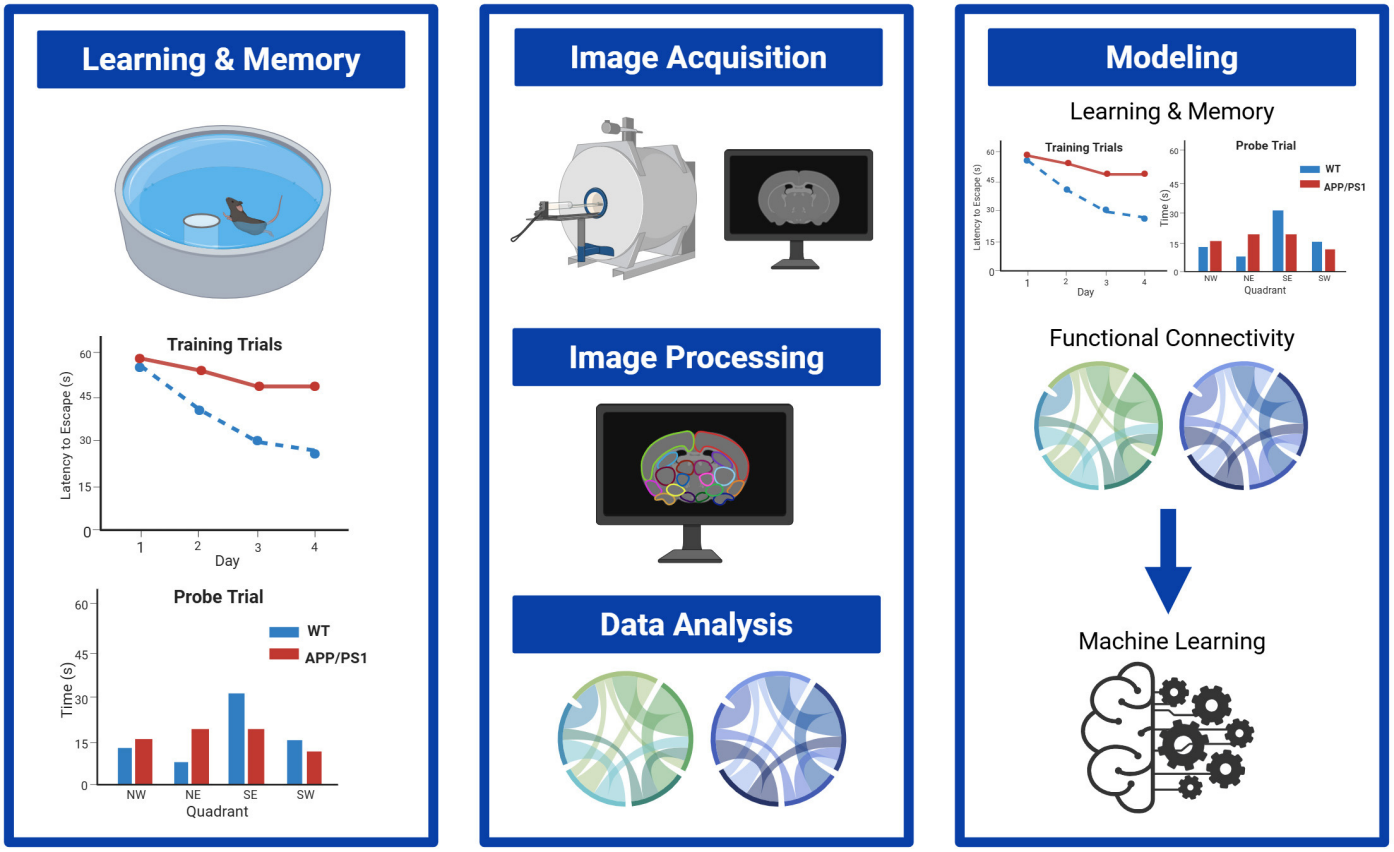
Overview of experimental approach. First, Morris Water Maze was conducted to test spatial learning and memory, followed by rs-fMRI in awake mice to measure functional connectivity changes. All assays were conducted in 3-, 6, and 10-month APP/PS1 mice and WT controls. Using a machine learning model, the relationships between learning and memory and functional connectivity were assessed. Created in BioRender, Hipskind, E. (2025) https://BioRender.com/mqc3bdg.

This study established changes in FC and investigated the relationship between FC and cognitive decline in an Aβ mouse model of AD, Cg-Tg(APPswe,PSEN1dE9)85Dbo (APP/PS1) throughout disease progression, including before plaque deposition, at the beginning stage of plaque deposition, and after significant plaque deposition throughout the brain. Importantly, we conducted all rs-fMRI studies in awake, unanesthetized mice to minimize the confounding factors associated with the use of anesthetic agents. Using a machine learning model, we defined the interactions between neuroimaging readouts with learning and memory deficits across disease progression in control mice and the APP/PS1 mouse model of AD (Figure 1). We provide novel insights into how FC changes can be used explain spatial learning and memory performance. Additionally, these neuroimaging readouts show diagnostic and clinical significance by providing a method for early disease detection.

## 2 Materials and methods

All experimental protocols were approved by the Institutional Animal Care and Use Committee (IACUC) at Baylor College of Medicine and were in accordance with the guidelines published in the National Institutes of Health Guide for Care and Use of Laboratory Animals. Animals were maintained in standard housing conditions with a 12-h light/dark cycle and received food and water *ad libitum* throughout the study. The mice used for this study were purchased from the Jackson Laboratory (strain B6;C3-Tg(APPswe,PSEN1dE9)85Dbo/Mmjax; #34832). These mice are double transgenic mice co-expressing a chimeric mouse/human amyloid precursor protein harboring the Swedish K670M/N671L mutations (Mo/HuAPP695swe) and human presenilin 1 with the exon-9 deletion mutation (PS1dE9), and they begin to show plaque deposition at about 6 months of age. Wild type (WT) mice consisted of the C57BL/6 background strain. Both male and female mice were used in the study.

### 2.1 Experimental design and statistical analyses

#### 2.1.1 Morris Water Maze

Spatial learning and memory were assessed using the Morris Water Maze assay. Briefly, mice completed 4 days of training trials and a probe trial on the final day. Mice were acclimated in the testing room for 30 min before the start of the experiment. On Day 1, mice underwent a training session before their first trial wherein they were briefly placed on the platform, tested to insure they could climb onto the hidden platform, and allowed to swim briefly (10 s) around the pool. Mice performed 2 blocks of 4 trials per day (60 s maximum per trial with 1-h inter-block interval) for 4 consecutive days. Latency to escape was recorded for each trial. On Day 4, after all training trials were completed, mice performed a probe trial, in which the hidden platform was removed, and mice were allowed to swim for 60 s. The time spent in each quadrant was recorded in the probe trial. The time spent in the target quadrant (percent of total time) was reported. The animals' trajectories were recorded with a video tracking system (Noldus EthoVision, Wageningen, the Netherlands). A 2-way ANOVA was used to compare WT and APP training and probe trial data. Significant results were followed by Tukey's *post-hoc t*-test.

### 2.2 Acclimation for awake imaging

Mice underwent a five-day conditioning paradigm to acclimate them to the awake imaging mouse holder, environment, and scanner sounds. Mice were briefly anesthetized with 0.5–1% isoflurane and placed in the restraint holder in an acclimation chamber, which is designed to mimic the environment of the MR scanner. Delivery of 0.5–1% isoflurane continued until mice were fully secured into the holder, then discontinued. Mice were given a minimum of 5 min to become fully awake (based on respiration rates returning to physiologically normal levels). On day one of acclimation, animals spent 10 min fully awake in the holder. Ten additional minutes were added each subsequent day, so that on day five of acclimation, mice remained in the holder for 50 min. During acclimation, respiration, heart rate, and temperature were monitored (Kent Scientific, Torrington, CT USA) and maintained at 37°C with an air-heating system. Following acclimation, mice were again briefly anesthetized for removal from the holder (Fadel et al., 2022).

### 2.3 Magnetic resonance imaging

All images were collected in awake, unanesthetized mice. As described in the acclimation protocol, mice were briefly anesthetized with 0.5–1% isoflurane and placed in the restraint holder in the scanner. After cessation of isoflurane, image acquisition began a minimum of 5 min later to ensure the mice were fully awake, as indicated by their respiration rates returning to physiologically normal levels (Fadel et al., 2022). Respiration, heart rate, and temperature were monitored during image acquisition using an MRI compatible small animal monitoring system (SA Instruments, Inc, Stony Brook, NY, USA). All images in this study were acquired with a 9.4T Bruker Advance III 20 cm bore MRI. rs-fMRI was used to measure functional connectivity between pre-selected anatomically defined brain regions. rs-fMRI data was acquired using EPI as a sequence of 300 volumes at 1.5 second intervals. EPI volumes were recorded at 0.25 x 0.25 x 1.25 mm voxel dimensions. A corresponding T2 image volume was also acquired at higher resolution. The T_2_-weighted imaging parameters were TR (rep time) = 2,500 ms, TE (echo time) = 11 ms, slice thickness = 1 mm, matrix = 256 × 256, spatial resolution = 0.156 mm/pixel, and number of averages = 1.

#### 2.3.1 Pre-processing

The time sequence was subjected to motion correction by alignment and then slice timing correction using SPM12. The output from alignment was checked prior to further processing and scans with excessive motion were discarded and a new scan was taken. The T2 volume and a mean of the EPI sequence were masked using Slicer (Fedorov et al., 2012) and the mask applied to the entire sequence. The T2 and EPI volumes were registered initially to a T2 template volume (Chon et al., 2019) using three successive iterations of the FSL flirt command with 6, 9, and 12 parameters respectively (rigid body, traditional, and affine registrations) (Jenkinson et al., 2002, 2012; Smith et al., 2004; Woolrich et al., 2009). A final co-registration was completed using FSL fnirt to the same template for the T2 volume and to an EPI template from Shella Keilholz (Xu et al., 2023). The final registrations were sampled isotropically at 200 uM resolution. The templates were in the coordinates of the Allen Brain Atlas padded with excess voxels in each dimension. To facilitate preprocessing, the steps were scripted to run automatically on a cohort of mice using MatLab running under Windows 10. The quality of the registrations was checked manually prior to denoising and analysis. ROI maps were derived from the Allen Brain Atlas using the data from the Allen Scalable Brain Atlas (Lein et al., 2007). Using MatLab scripting, semi-automated combination of the detailed regions specified in the scalable atlas was used to generate several distinct ROI maps that were focused on sets of relevant ROIs (see Table 1).

**Table 1:** rs-fMRI regions of interest.

**Region name**
Posterior parietal
Anterior cingulate
Infralimbic
Insula
Frontal Pole
Primary somatosensory
Visual
Temporal
Orbital Cortex
Retrospenial
Ectorhinal
Perirhinal
Prelimbic
Primary motor
Secondary motor
Auditory
Gustatory
Olfactory
Piriform
Hippocampus
Dentate gyrus
Subiculum
Entorhinal
Cortical subplate
Striatum
Pallidulum
Thalamus
Hypothalamus
Midbrain
Pons

30 regions selected from the Allen Brain Mouse Reference Atlas. Functional Connectivity was assessed between all regions.

#### 2.3.2 Mouse connectomes

Results included in this manuscript come from analyses performed using CONN (Whitfield-Gabrieli and Nieto-Castanon, 2012) (RRID:SCR_009550) release 22.a (Nieto-Castanon and Whitfield-Gabrieli, 2022) and SPM (Penny et al., 2011) (RRID:SCR_007037) release 12.7771.

##### 2.3.2.1 Denoising

Functional data were denoised using a standard denoising pipeline (Nieto-Castanon, 2020) including the regression of potential confounding effects characterized by white matter timeseries (5 CompCor noise components), CSF timeseries (5 CompCor noise components), session effects and their first order derivatives (2 factors), and linear trends (2 factors) within each functional run, followed by bandpass frequency filtering of the BOLD timeseries (Hallquist et al., 2013) between 0.008 Hz and 0.09 Hz. CompCor (Behzadi et al., 2007; Chai et al., 2012) noise components within white matter and CSF were estimated by computing the average BOLD signal as well as the largest principal components orthogonal to the BOLD average within each subject's eroded segmentation masks.

##### 2.3.2.2 First-level analysis

ROI-to-ROI connectivity (RRC) matrices were estimated characterizing the functional connectivity between each pair of regions among 30 Allen Brain Atlas ROIs (Table 1) (60 bilateral regions). For bilateral results see Supplementary Tables 1–3 (Desikan et al., 2006). Functional connectivity strength was represented by Fisher-transformed bivariate correlation coefficients from a general linear model (weighted-GLM) (Nieto-Castanon, 2020), estimated separately for each pair of ROIs, characterizing the association between their BOLD signal timeseries.

A 2-way ANOVA was used to assess the effects of age and genotype (APP/PS1 and wild-type). Each connection was analyzed using the MatLab *anovan* function and yielded *p*-values for effects of WT vs. APP/PS1, for aging, and an interaction between the two. The *p*-values were sorted and a false discovery rate calculation was used to determine the most significant values.

##### 2.3.2.3 Group-level analyses

Group-level analyses were performed using a General Linear Model (GLM) (Nieto-Castanon, 2020). For each individual connection a separate GLM was estimated, with first-level connectivity measures at this connection as dependent variables (one independent sample per subject and one measurement per task or experimental condition, if applicable), and groups or other subject-level identifiers as independent variables. Connection-level hypotheses were evaluated using multivariate parametric statistics with random-effects across subjects and sample covariance estimation across multiple measurements. Inferences were performed at the level of individual functional connections, and results were thresholded using a familywise corrected *p*-FDR < 0.01 connection-level threshold (Benjamini and Hochberg, 1995; Nieto-Castanon, 2020). Clustering of brain regions in cord diagrams was determined by anatomical proximity and functional similarity, with the 3-month time point used as a base line and applied to the other time points.

### 2.4 Machine learning

We created linear regression models of Morris Water Maze (MWM) data using ROI-to-ROI connectivity data (*p* < 0.05) as the predictor (independent variables) and memory performance as the dependent variable. Specifically, we modeled the relationship between learning using the MWM training trial data (latency to escape on Day 1–Day 4) and functional connectivity. A second model using the MWM probe trail data (time spent in SE quadrant) was created to model the relationship between memory performance and functional connectivity. Separate models were trained for each time point for the APP/PS1 mice. Modeling was performed using the 6- and 10-month APP/PS1 data. These time points were chosen because they showed spatial memory deficits, whereas the 3-month time point does not exhibit changes in spatial memory performance. At a given time point, the MWM training data (Model 1) or probe trial date (Model 2) (*y*) of a mouse was fit as:


$$\begin{array}{l} y=\beta_{0}+\overset{i=n}{\underset{i=1}{\sum }}\beta_{i}x_{i} \end{array}$$

where each *x*_*i*_ denotes the connectivity data between a pair of ROIs. The *n* ROI pairs used in the model were chosen using the connections that had significant differences in functional connectivity (see Conn analysis above). Variables were selected based on the significant connection-level results (p < 0.05) from the group-level analysis performed in Conn analyzing ROI-to-ROI FC from each time point and filtered to DMN and regions known to be highly involved in memory function (cite). This was done to avoid over fitting and to provide the most biologically relevant results. To facilitate a comparison between β coefficients of different variables, each variable's values were linearly scaled between 0 and 1. We then fit a Lasso-regularized model, meaning that we estimated the values of the β parameters above by minimizing the following error function:


$$\begin{array}{l} {\left(y-\left(\beta_{0}+\overset{i=n}{\underset{i=1}{\sum }}\beta_{i}x_{i}\right)\right)}^{2}+\alpha \overset{i=n}{\underset{i=0}{\sum }}|\beta_{i}| \end{array}$$

where α is the hyperparameter. For values of α>0, this error function penalizes the model for using more variables than would be necessary to explain the variance in the data. The penalty increases with increasing values of α. Thus, the Lasso-regularized error function guards against overfitting by constraining the model to utilize as few predictor variables as possible (Ranstam and Cook, 2018). As additional rigor, we estimated the value of α using leave-one-out cross-validation (LOOCV). It has been shown theoretically that LOOCV provides an unbiased estimate of α (Sivula et al., 2022).

## 3 Results

### 3.1 Morris Water Maze

**Figure 2:**
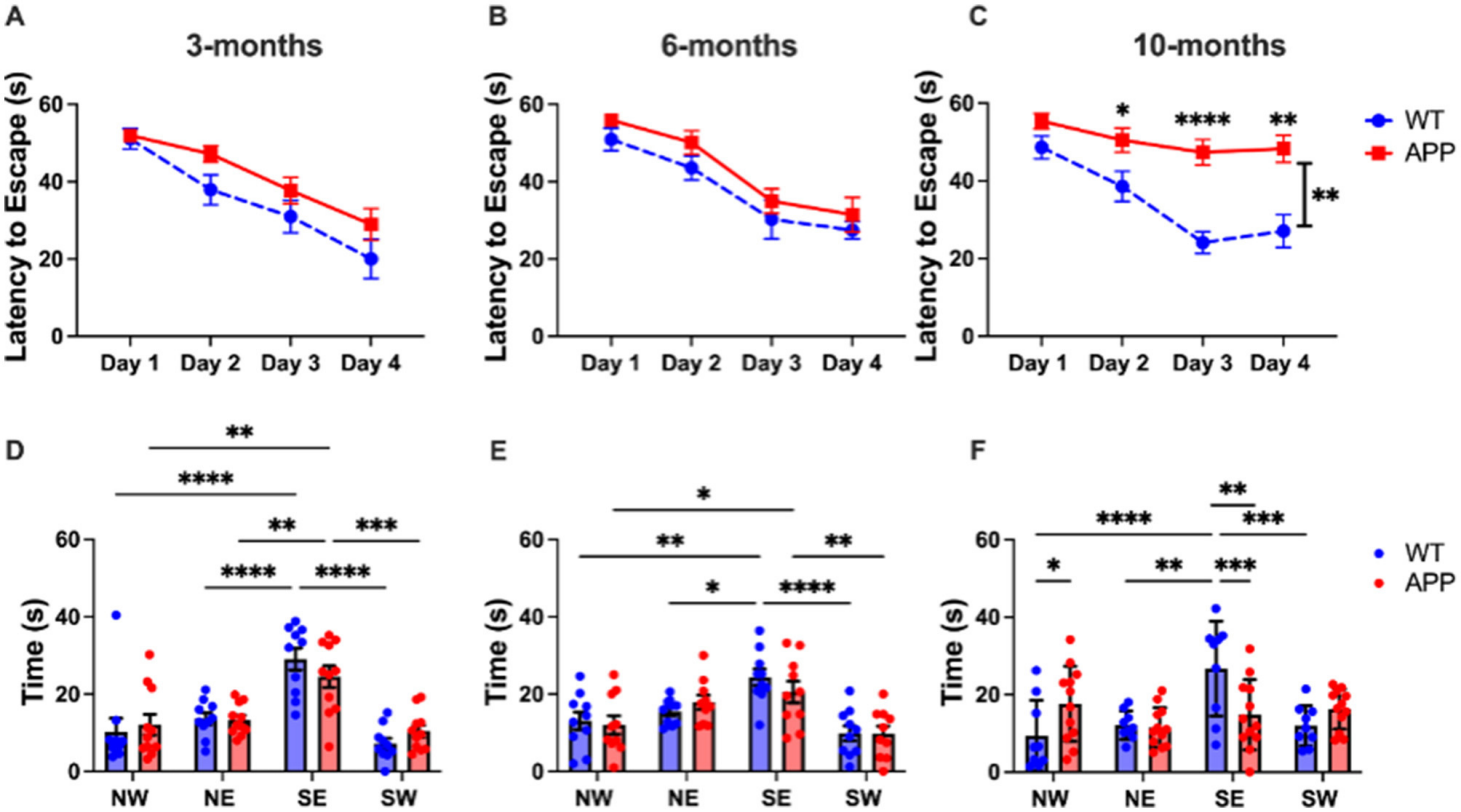
10-month APP/PS1 mice showed deficits in spatial learning and memory. In the training trials of the Morris Water Maze assay, 3- and 6-month-old mice displayed normal learning **(A, B)**, while 10-month-olds exhibited significant deficits (*p* < 0.01) **(C)**. 3-month-old mice showed no deficits in the MWM probe trials **(D)**. At 6 months, memory deficits emerged, where APP/PS1 do not differentiate between NE and SE zones **(E)**. By 10 months, APP/PS1 mice exhibited no spatial preference for the SE zone **(F)**. All figures show mean ± SEM, individual points depict a single mouse. 3 months: APP/PS1 *N* = 11, WT *N* = 10, 6 months: APP/PS1 *N* = 10, WT *N* = 10, 10 months: APP/PS1 *N* = 12, WT *N* = 9. *p < 0.05, **p < 0.01, ***p < 0.001, ****p < 0.0001.

Morris Water Maze was used to assess spatial learning and memory. The Morris Water Maze is preferred for its ability to quantitatively assess spatial learning and memory with high reliability and minimal stress to the animals, providing consistent and objective data relevant to human cognitive disorders. During the training phase, mice learned to find a hidden platform in a pool of water over 4 days of training, measured by latency to escape. At 3 months of age, a 2-way ANOVA showed a significant effect of Day [F_(3, 76)_ = 21.77, *p* < 0.0001], but not Genotype [F_(1, 19)_ = 2.956, *p* = 0.1018], and no significant interaction between Day and Genotype [F_(3, 57)_ = 1.088, *p* = 0.3614], indicating no significant difference in learning (Figure 2A). Similarly for 6 months, there was a significant effect of Day [F_(2.442, 43.96)_ = 35.15, *p* < 0.0001], but not Genotype [F_(1, 18)_ = 2.258, *p* = 0.1503], and no interaction [F_(3, 54)_ = 0.07746, *p* = 0.9719] (Figure 2B). At 10-months, there was a significant effect of Day [F_(2.633, 50.03)_ = 20.95, *p* < 0.00010], Genotype [F_(1, 19)_ = 18.30, *p* = 0.0004], and interaction [F_(3, 57)_ = 5.815, *p* = 0.0015] (Figure 2C). *Post-hoc* Tukey's t-test with multiple comparisons correction revealed a significant difference between the APP/PS1 and WT latency to escape on Day 2 (*p* = 0.0297), Day 3 (*p* < 0.0001), and Day 4 (*p* = 0.0012).

A final probe trial was used to assess spatial memory. At 3 months, a 2-way ANOVA revealed a significant effect of Quadrant [F_(3, 72)_ = 13.70, *p* < 0.0001], but not Genotype [F_(1, 76)_ = 2.355e-006, *p* = 0.9988], with no significant interaction (Figure 2D). Post-hoc Tukey's *t*-tests with multiple comparisons correction revealed that both APP/PS1 and WT mice spent significantly more time in the SE quadrant (Figure 2D) where the platform was previously located, indicating that both groups successfully remembered the platform location. Additionally, there was no significant difference in time spent in SE quadrant between APP/PS1 and WT (*p* = 0.1618).

At 6 months, a 2-way ANOVA revealed a significant effect of Quadrant [F_(3, 76)_ = 24.33, < 0.0001], but not Genotype [F_(1, 72)_ = 0.1910, *p* = 0.6634], and no significant interaction [F_(3, 72)_ = 0.7389, *p* = 0.5323] (Figure 2E). Based on the *post-hoc* Tukey's *t*-test with multiple comparisons correction, there is evidence of the beginning of memory deficits in the 6-month APP/PS1 mice (Figure 2E). While there was no significant difference in time spent in SE quadrant between APP/PS1 and WT (*p* = 0.2056), the APP/PS1 had no significant difference between time spent in the SE and NE quadrant, suggesting the beginning of memory deficits.

At 10 months, a 2-way ANOVA revealed a significant effect of Quadrant [F_(3, 76)_ = 5.138, *p* = 0.0027], but not Genotype [F_(1, 76)_ = 6.976e-012, *p* > 0.9999]; however, there was a significant interaction [F_(3, 76)_ = 6.342, *p* = 0.0007] (Figure 2F). The *post-hoc* Tukey's *t*-test with multiple comparisons correction revealed a significant difference in time spent in SE quadrant between APP/PS1 and WT (*p* = 0.0010). The APP/PS1 mice showed no significant difference between time spent in any quadrant (*p* > 0.05).

### 3.2 Functional connectivity

**Figure 3:**
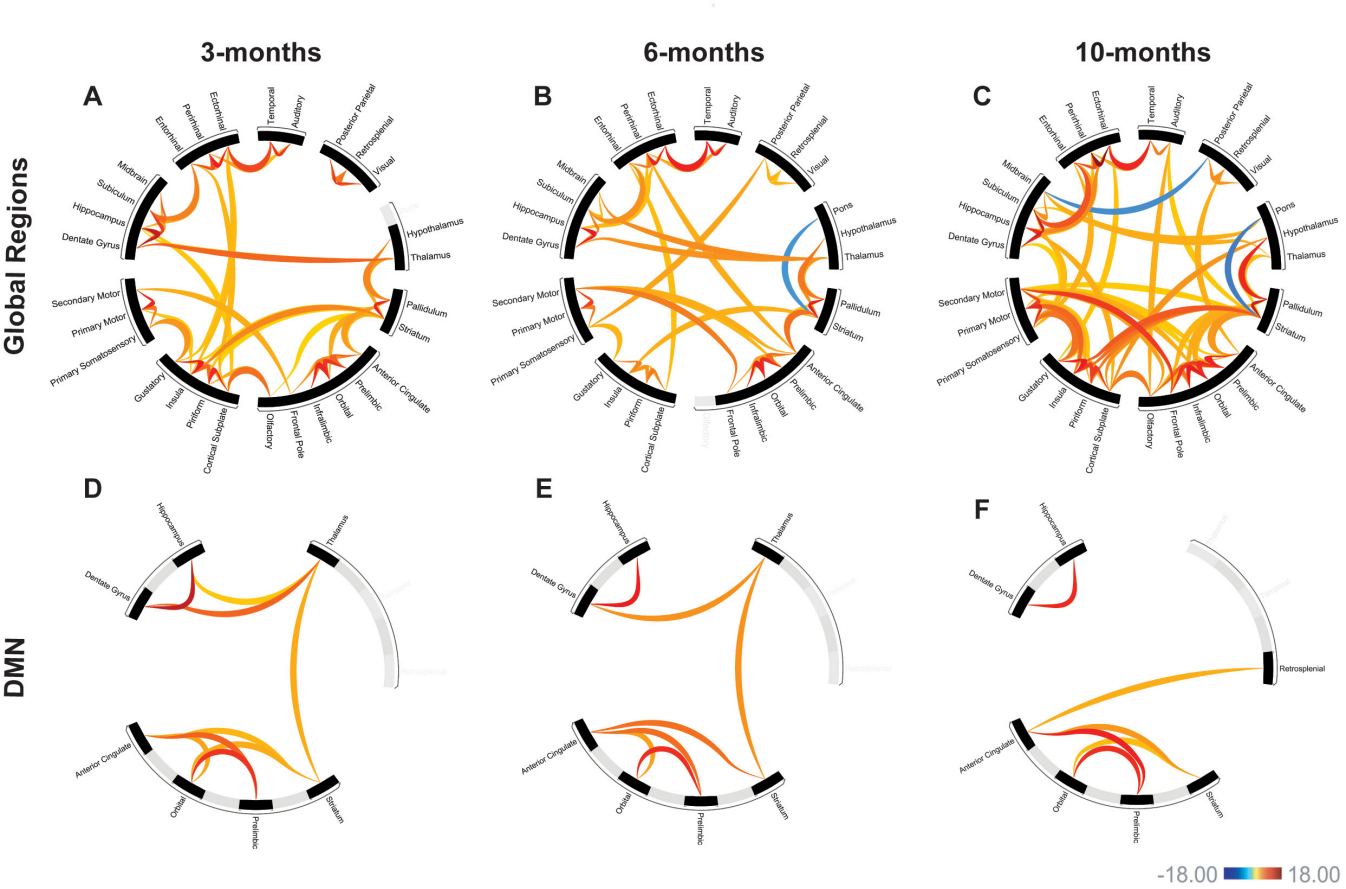
APP/PS1 mice exhibited progressive hyperconnectivity. Functional connectivity in APP/PS1 mice compared to WT. Warm colors represent hyperconnectivity, cool colors indicate hypoconnectivity. Over all 30 assessed regions, APP/PS1 mice exhibit primarily hyperconnectivity **(A–C)**. This hyperconnectivity was present by 3-months **(A)**, with progressive increases in over 6- and 10-months **(B, C)**. In the default-mode network (DMN), there was also evidence of early hyperconnectivity at 3-months **(D)**. Similar changes in connectivity were seen at 6- and 10-months **(E, F)**. Scale bar represents z-score, only significant connections are shown. Linear regression model, all comparisons APP/PS1>WT. 3-months: WT *N* = 8, APP/PS1 *N* = 10; 6-months: WT *N* = 10, APP/PS1 *N* = 10; 10-months: WT *N* = 10, APP/PS1 *N* = 12.

Functional connectivity was assessed between 30 regions of interest (435 total connections) in the APP/PS1 mice compared to WT. We primarily observed hyperconnectivity in the APP/PS1 mice across all time points. A 2-way ANOVA revealed a significant effect of age (see Supplementary Table 4 for detailed statistics). At 3 months, we observed hyperconnectivity in 47 ROI-to-ROI connections (Figure 3A) (Table 2). This included memory related regions such as the hippocampus, dentate gyrus, entorhinal cortex, and the cortical sublate. At 6 months, 45 connections exhibited hyperconnectivity, while hypoconnectivity was seen in one connection (between the striatum and pons) (Figure 3B, Table 3). By 10 months, 84 regions were hyperconnected (Figure 3C, Table 4). The striatum and pons continued to exhibit hypoconnectivity, and hypoconnectivity emerged between the midbrain and posterior parietal cortex. There were several connections (32 connections) that exhibited hyperconnectivity across all time points in the APP/PS1 mice compared to WT. Several of these included the ectorhinal cortex and temporal cortex, entorhinal cortex and ectorhinal cortex, hippocampus and dentate gyrus, striatum and anterior cingulate cortex, cortical subplate and piriform cortex, thalamus and pallidum, insula and piriform cortex, insula and striatum, dentate gyrus and subiculum, orbital cortex and prelimbic cortex, orbital cortex and infralimbic cortex. See Tables 2–4 for complete list and detailed statistics.

**Table 2:** 3-month functional connectivity changes in APP/PS1 compared to WT.

**Region 1**	**Region 2**	**T(df)**	***p* FDR**
^*^Hippocampus	Dentate gyrus	T(16) = 14.59	< 0.0000001
^*^Gustatory	Insula	T(16) = 11.92	< 0.0000001
^*^Ectorhinal area	Perirhinal	T(16) = 11.26	< 0.0000001
^*^Striatum	Pallidulum	T(16) = 11.23	0.000001
^*^Infralimbic	Orbital	T(16) = 11.06	0.000001
^*^Secondary motor	Primary motor	T(16) = 10.73	0.000001
^*^Piriform	Cortical Subplate	T(16) = 10.44	0.000001
^*^Orbital	Prelimbic	T(16) = 9.94	0.000002
^*^Auditory	Temporal	T(16) = 9.68	0.000002
^*^Visual	Posterior parietal	T(16) = 9.57	0.000002
^*^Subiculum	Dentate gyrus	T(16) = 9.52	0.000002
^*^Temporal	Ectorhinal	T(16) = 8.79	0.000006
^*^Perirhinal area	Entorhinal	T(16) = 8.43	0.000009
^*^Prelimbic area	Anterior cingulate	T(16) = 7.56	0.000035
Thalamus	Dentate gyrus	T(16) = 7.4	0.000044
^*^Insula	Piriform	T(16) = 7.29	0.000049
^*^Midbrain	Subiculum	T(16) = 7.18	0.000056
Cortical Subplate	Olfactory	T(16) = 7.04	0.000068
^*^Visual	Retrosplenial	T(16) = 6.77	0.000104
^*^Primary motor	Primary somatosensory	T(16) = 6.61	0.00013
^*^Infralimbic	Prelimbic	T(16) = 6.55	0.00014
^*^Retrosplenial	Posterior parietal	T(16) = 6.5	0.000146
^*^Frontal pole	Prelimbic	T(16)= 6.37	0.000176
^*^Entorhinal	Subiculum	T(16) = 6.29	0.000196
^*^Insula	Striatum	T(16) = 6.02	0.0003
^*^Pallidulum	Hypothalamus	T(16) = 6.02	0.0003
^*^Infralimbic	Anterior cingulate	T(16) = 5.86	0.000389
^*^Primary somatosensory	Gustatory	T(16) = 5.43	0.00086
Midbrain	Dentate gyrus	T(16) = 5.31	0.001046
^*^Temporal	Perirhinal	T(16) = 5.28	0.001071
^*^Pallidulum	Thalamus	T(16) = 5.27	0.001071
Anterior cingulate	Pallidulum	T(16) = 5.19	0.001213
^*^Insula	Cortical Subplate	T(16) = 4.99	0.001746
Orbital	Anterior cingulate	T(16) = 4.87	0.002187
Orbital	Striatum	T(16) = 4.83	0.002298
Gustatory	Piriform	T(16) = 4.77	0.002469
Striatum	Thalamus	T(16) = 4.77	0.002469
^*^Secondary motor	Frontal pole	T(16)= 4.69	0.002816
Piriform	Olfactory	T(16) = 4.66	0.002929
^*^Entorhinal	Hippocampus	T(16) = 4.6	0.003207
Ectorhinal	Entorhinal	T(16) = 4.55	0.003501
^*^Anterior cingulate	Striatum	T(16) = 4.34	0.005144
Cortical Subplate	Entorhinal	T(16) = 4.34	0.005144
Insula	Ectorhinal	T(16) = 4.32	0.005252
Auditory	Ectorhinal	T(16) = 4.1	0.008037
Frontal pole	Orbital	T(16)= 4.07	0.008427
Gustatory	Ectorhinal	T(16) = 3.99	0.009716

^*^Denotes connections that altered across all time points.

Each row depicts a functional connection between two brain regions. Order of regions (Region 1 or Region 2) does not imply directionality of the connection. Only connections that have significant differences between APP/PS1 and WT mice are shown. All comparisons based on a linear regression model, APP/PS1 > WT. T is the t-statistic and df = degrees of freedom. FDR-corrected (p FDR) p-values are given.

**Table 3:** 6-month functional connectivity changes in APP/PS1 compared to WT.

**Region 1**	**Region 2**	**T(df)**	***p*-FDR**
^*^Striatum	Pallidulum	T(18) = 12.64	< 0.0000001
^*^Hippocampus	Dentate gyrus	T(18) = 11.98	< 0.0000001
^*^Auditory	Temporal	T(18) = 11.6	< 0.0000001
^*^Ectorhinal	Perirhinal	T(18) = 11.49	< 0.0000001
^*^Infralimbic	Orbital	T(18) = 11.15	< 0.0000001
^*^Temporal	Ectorhinal	T(18) = 11.08	< 0.0000001
^*^Orbital	Prelimbic	T(18) = 10.25	< 0.0000001
^*^Secondary motor	Primary motor	T(18) = 9.1	0.000002
^*^Prelimbic	Anterior cingulate	T(18) = 7.15	0.000056
^*^Anterior cingulate	Striatum	T(18) = 6.97	0.000071
^*^Pallidulum	Hypothalamus	T(18) = 6.64	0.000124
^*^Piriform	Cortical Subplate	T(18) = 6.53	0.000141
^*^Perirhinal	Entorhinal	T(18) = 6.43	0.000159
^*^Primary motor	Primary somatosensory	T(18) = 6.32	0.000175
^*^Secondary motor	Frontal pole	T(18) = 6.31	0.000175
^*^Subiculum	Dentate gyrus	T(18) = 6.22	0.000195
^*^Gustatory	Insula	T(18) = 6.06	0.000255
Thalamus	Midbrain	T(18) = 5.81	0.000401
^*^Entorhinal	Subiculum	T(18) = 5.65	0.000514
Striatum	Thalamus	T(18) = 5.62	0.000514
Thalamus	Dentate gyrus	T(18) = 5.62	0.000514
^*^Insula	Piriform	T(18) = 5.59	0.000526
Auditory	Ectorhinal	T(18) = 5.4	0.000751
Orbital	Anterior cingulate	T(18) = 5.07	0.001386
^*^Frontal pole	tex Prelimbic	T(18) = 5.05	0.0014
Primary somatosensory	Posterior parietal	T(18) = 4.86	0.002008
^*^Temporal	Perirhinal	T(18) = 4.81	0.002192
^*^Pallidulum	Thalamus	T(18) = 4.74	0.002442
Anterior cingulate	Perirhinal	T(18) = 4.65	0.002808
Secondary motor	Anterior cingulate	T(18) = 4.65	0.002808
^*^Visual	Posterior parietal	T(18) = 4.58	0.00313
^*^Insula	Striatum	T(18) = 4.52	0.003513
^*^Infralimbic	Prelimbic	T(18) = 4.49	0.003623
^*^Insula	Cortical Subplate	T(18) = 4.46	0.003735
^*^Midbrain	Subiculum	T(18) = 4.44	0.003823
^*^Retrosplenial	Posterior parietal	T(18) = 4.41	0.004007
Secondary motor	Prelimbic	T(18) = 4.39	0.004087
^*^Primary somatosensory	Gustatory	T(18) = 4.37	0.004107
^*^Infralimbic	Anterior cingulate	T(18) = 4.29	0.004817
^*^Entorhinal	Hippocampus	T(18) = 4.2	0.005714
Cortical Subplate	Entorhinal	T(18) = 4.15	0.006205
Perirhinal	Hippocampus	T(18) = 4.14	0.006205
Ectorhinal	Hippocampus	T(18) = 4.1	0.006647
Anterior cingulate	Pallidulum	T(18) = 4.02	0.007805
^*^Visual	Retrosplenial	T(18) = 3.98	0.008278
Striatum	Pons	T(18) = −5.27	0.000952

^*^Denotes connections that altered across all time points.

Each row depicts a functional connection between two brain regions. Order of regions (Region 1 or Region 2) does not imply directionality of the connection. Only connections that have significant differences between APP/PS1 and WT mice are shown. All comparisons based on a linear regression model, APP/PS1 > WT. T is the t-statistic and df=degrees of freedom. FDR-corrected (p FDR) p-values are given.

**Table 4:** 10-month functional connectivity changes in APP/PS1 compared to WT.

**Region 1**	**Region 2**	**T(df)**	***p*-FDR**
^*^Ectorhinal	Perirhinal	T(18) = 17.26	< 0.0000001
^*^Striatum	Pallidulum	T(18) = 14.35	< 0.0000001
^*^Subiculum	Dentate gyrus	T(18) = 14	< 0.0000001
^*^Infralimbic	Orbital	T(18) = 12.61	< 0.0000001
^*^Gustatory	Insula	T(18) = 12.04	< 0.0000001
^*^Prelimbic	Anterior cingulate	T(18) = 11.38	< 0.0000001
^*^Primary motor	Primary somatosensory	T(18) = 10.75	< 0.0000001
^*^Pallidulum	Hypothalamus	T(18) = 10.65	< 0.0000001
^*^Hippocampus	Dentate gyrus	T(18) = 10.63	< 0.0000001
^*^Temporal	Ectorhinal	T(18) = 10.6	< 0.0000001
^*^Orbital	Prelimbic	T(18) = 10.32	< 0.0000001
Frontal pole	Orbital	T(18) = 10.15	< 0.0000001
^*^Secondary motor	Frontal pole	T(18) = 9.43	0.000001
^*^Infralimbic	Prelimbic	T(18) = 9.4	0.000001
^*^Piriform	Cortical subplate	T(18) = 9.37	0.000001
^*^Insula	Piriform	T(18) = 9.18	0.000001
^*^Secondary motor	Primary motor	T(18) = 8.98	0.000001
^*^Frontal pole	Prelimbic	T(18) = 8.53	0.000002
^*^Perirhinal	Entorhinal	T(18) = 8.23	0.000004
^*^Entorhinal	Subiculum	T(18) = 8.02	0.000005
Gustatory	Striatum	T(18) = 7.97	0.000005
Olfactory	Orbital	T(18) = 7.79	0.000007
Primary motor	Insula	T(18) = 7.34	0.000015
^*^Insula	Striatum	T(18) = 7.19	0.00002
^*^Primary somatosensory	Gustatory	T(18) = 6.84	0.000037
Primary motor	Gustatory	T(18) = 6.7	0.000047
Olfactory	Infralimbic	T(18) = 6.6	0.000054
Piriform	Striatum	T(18) = 6.53	0.000061
Cortical subplate	Olfactory	T(18) = 6.49	0.000063
Auditory	Temporal	T(18) = 6.33	0.000084
Gustatory	Piriform	T(18) = 5.95	0.000167
^*^Ectorhinal	Hippocampus	T(18) = 5.94	0.000167
^*^Insula	Cortical subplate	T(18) = 5.83	0.000203
^*^Retrosplenial	Posterior parietal	T(18) = 5.72	0.000249
Cortical subplate	Striatum	T(18) = 5.67	0.000268
^*^Midbrain	Subiculum	T(18) = 5.66	0.000269
^*^Visual	Retrosplenial	T(18) = 5.44	0.000406
Cortical subplate	Hypothalamus	T(18) = 5.43	0.000406
Posterior parietal	Midbrain	T(18) = 5.43	0.000406
^*^Infralimbic	Anterior cingulate	T(18) = 5.41	0.000413
^*^Anterior cingulate	Striatum	T(18) = 5.38	0.00043
Primary somatosensory	Insula	T(18) = 5.34	0.000452
Secondary motor	Olfactory	T(18) = 5.1	0.000736
^*^Visual	Posterior parietal	T(18) = 5.09	0.000745
Entorhinal	Dentate gyrus	T(18) = 5.04	0.000799
^*^Entorhinal	Hippocampus	T(18) = 5	0.000851
^*^Temporal	Perirhinal	T(18) = 4.98	0.000883
Frontal pole	Tex infralimbic	T(18) = 4.95	0.000926
Infralimbic	Striatum	T(18) = 4.86	0.001085
Orbital	Pallidulum	T(18) = 4.74	0.001398
Cortical subplate	Hippocampus	T(18) = 4.72	0.001434
Thalamus	Midbrain	T(18) = 4.71	0.001434
Pons	Midbrain	T(18) = 4.7	0.00144
Anterior cingulate	Retrosplenial	T(18) = 4.62	0.001656
Olfactory	Auditory	T(18) = 4.61	0.001656
Secondary motor	Orbital	T(18) = 4.61	0.001656
Primary motor	Piriform	T(18) = 4.59	0.00169
Olfactory	Prelimbic	T(18) = 4.56	0.001761
Primary somatosensory	Entorhinal	T(18) = 4.56	0.001761
Olfactory	Frontal pole	T(18) = 4.43	0.002293
Secondary motor	Insula	T(18) = 4.42	0.002315
Olfactory	Striatum	T(18) = 4.37	0.002564
Perirhinal	Hippocampus	T(18) = 4.3	0.0029
Gustatory	Cortical subplate	T(18) = 4.29	0.002963
^*^Pallidulum	Thalamus	T(18) = 4.25	0.003133
Primary motor	Frontal pole	T(18) = 4.25	0.003133
Ectorhinal	Entorhinal	T(18) = 4.24	0.003133
Primary somatosensory	Subiculum	T(18) = 4.23	0.003198
Orbital	Striatum	T(18) = 4.18	0.003482
Secondary motor	Piriform	T(18) = 4.17	0.003559
Infralimbic	Pallidulum	T(18) = 4.12	0.003858
Ectorhinal	Dentate gyrus	T(18) = 4.11	0.003936
Secondary motor	Cortical subplate	T(18) = 4.01	0.00474
Secondary motor	Gustatory	T(18) = 4.01	0.00474
Gustatory	Ectorhinal	T(18) = 3.95	0.005362
Secondary motor	Primary somatosensory	T(18) = 3.94	0.005369
Pons	Subiculum	T(18) = 3.88	0.006031
Secondary motor	Anterior cingulate	T(18) = 3.88	0.006031
Olfactory	Pallidulum	T(18) = 3.87	0.006049
Striatum	Auditory	T(18) = 3.83	0.006542
Secondary motor	Striatum	T(18) = 3.8	0.006951
Secondary motor	Prelimbic	T(18) = 3.77	0.007272
Cortical subplate	Pallidulum	T(18) = 3.76	0.007365
Striatum	Hypothalamus	T(18) = 3.73	0.007768
Primary somatosensory	Dentate gyrus	T(18) = 3.64	0.009427
Striatum	Pons	T(18) = −6.07	0.000138

^*^Denotes connections that altered across all time points.

Each row depicts a functional connection between two brain regions. Order of regions (Region 1 or Region 2) does not imply directionality of the connection. Only connections that have significant differences between APP/PS1 and WT mice are shown. All comparisons based on a linear regression model, APP/PS1 > WT. T is the t-statistic and df=degrees of freedom. FDR-corrected (p FDR) p-values are given.

Functional connectivity between DMN-like regions was also assessed (Xu et al., 2022). We saw early hyperconnectivity in this network at 3-months, with 9 connections exhibiting hyperconnectivity compared to WT (Figure 3D). Interestingly, we did not see progressive hyperconnectivity in this network, as there were fewer hyperconnected connections (7) at the 6-month time point (Figure 3E). Specifically, the hippocampus-thalamus and orbital-striatum connections were lost from the 3-month cohort to the 6-month cohort. At 10-months, 6 connections exhibited hyperconnectivity (Figure 3F). We observed the emergence of hyperconnectivity between the anterior cingulate and retrosplenial cortex. Additionally, there was a loss of hyperconnectivity with the thalamus at 10-months. The hippocampus-dentate gyrus connection remained hyperconnected across time points, as well as several connections with anterior cingulate cortex. See Table 5 for detailed statistics.

**Table 5:** Default mode network functional connectivity.

**3 month DMN**		**T(df)**	***p* FDR**
**Region 1**	**Region 2**		
Hippocampus	Dentate gyrus	T(16) = 14 59	< 0.0000001
Orbital	Prelimbic	T(16) = 9 94	0.000001
Anterior cingulate	Prelimbic	T(16) = 7 56	0.000014
Thalamus	Dentate gyrus	T(16) = 7 40	0.000014
Anterior cingulate	Orbital	T(16) = 4 87	0.00108
Orbital	Striatum	T(16) = 4 83	0.00108
Striatum	Thalamus	T(16) = 4 77	0.00108
Anterior cingulate	Striatum	T(16) = 4 34	0.002288
Thalamus	Hippocampus	T(16) = 3 88	0.00535
**6 month DMN**			
Hippocampus	Dentate gyrus	T(18) = 11 98	< 0.0000001
Orbital	Prelimbic	T(18) = 10 25	< 0.0000001
Anterior cingulate	Prelimbic	T(18) = 7 15	0.000014
Anterior cingulate	Striatum	T(18) = 6 97	0.000015
Striatum	Thalamus	T(18) = 5 62	0.000149
Thalamus	Dentate gyrus	T(18) = 5 62	0.000149
Anterior cingulate	Orbital	T(18) = 5 07	0.00041
**10 month DMN**			
Anterior cingulate	Prelimbic	T(18) = 11 38	< 0.0000001
Hippocampus	Dentate gyrus	T(18) = 10 63	< 0.0000001
Orbital	Prelimbic	T(18) = 10 32	< 0.0000001
Anterior cingulate	Striatum	T(18) = 5 38	0.000374
Anterior cingulate	Retrosplenial	T(18) = 4 62	0.001545
Orbital	Striatum	T(18) = 4 18	0.003362

Changes in functional connectivity in the DMN at the 3, 6-, and 10-month in APP/PS1 compared to WT. Each row depicts a functional connection between two brain regions. Order of regions (Region 1 or Region 2) does not imply directionality of the connection. Only connections that have significant differences between APP/PS1 and WT mice are shown. All comparisons based on a linear regression model, APP/PS1 > WT. Both uncorrected (p unc) and FDR-corrected (p FDR) p-values are given.

### 3.3 Machine learning

**Figure 4:**
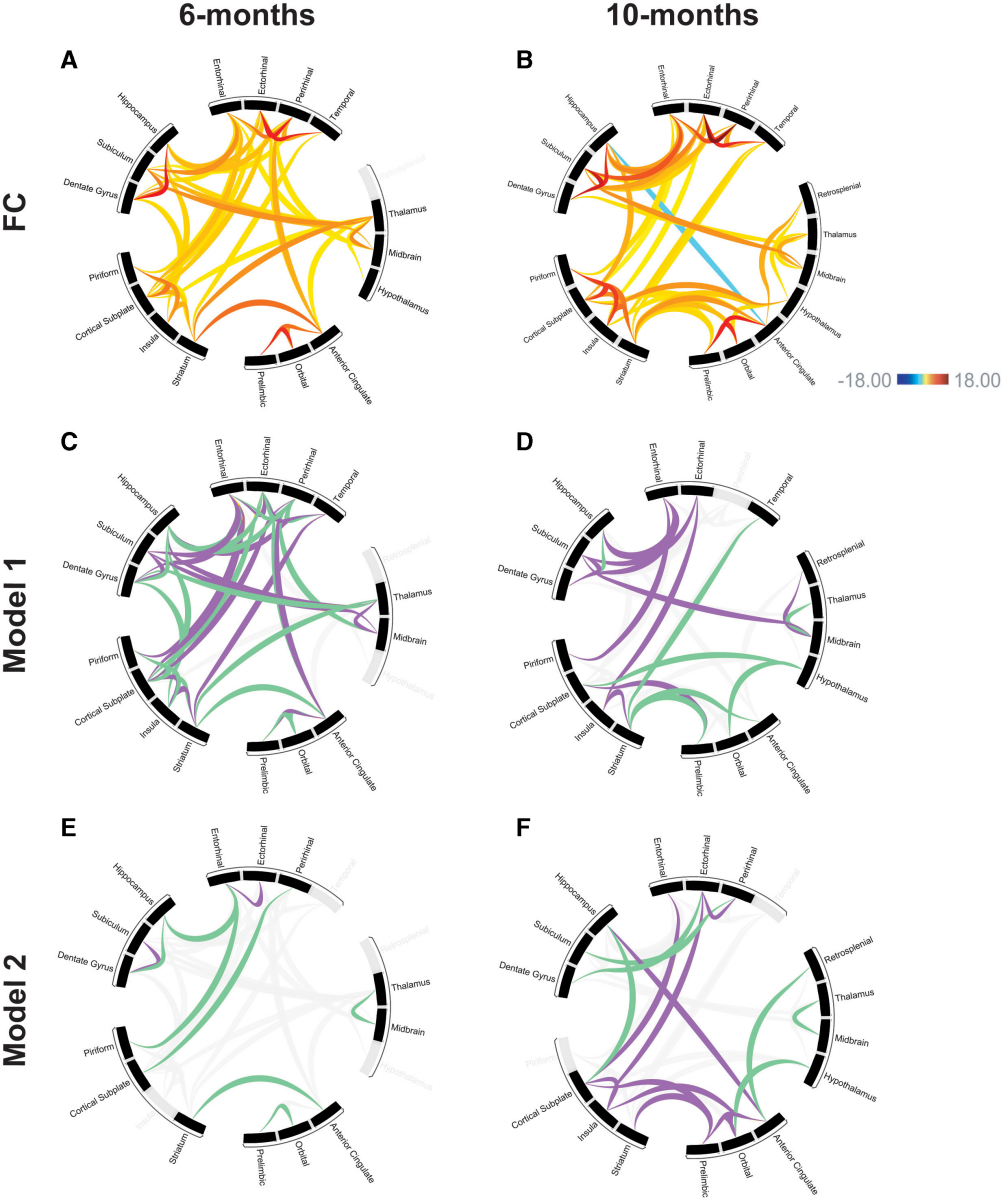
Modeling the relationships between functional connectivity and memory performance. Functional connectivity at 6-months **(A)** and 10-months **(B)** for DMN and memory-related brain regions used in subsequent modeling. Scale bar represents z-score, only significant connections are shown (*p* < 0.05). Linear regression model, all comparisons APP/PS1>WT. Model 1: relationship between MWM training data (spatial learning) and functional connectivity in the 6-month **(C)** and 10-month **(D)** groups. Model 2: relationship between MWM probe trial (spatial memory) and functional connectivity in the 6-month **(E)** and 10-month **(F)** groups. Purple and green connections have significant explanatory power for behavior based on the modeling, connections shown in gray were not predictive of behavioral performance. Purple: positive model coefficient, connection strength has a positive correlation with memory performance. Green: negative model coefficient, connection strength has a negative correlation with memory performance.

Prior to training the models, variable selection was performed to avoid overfitting. Variables were selected based on the significant connection-level results (p < 0.05) from the FC analysis. Regions were filtered to DMN and regions known to be highly involved in memory function (Figures 4A, B), which improved model fit [see Supplementary Table 5 for Akaike Information Criterion (AIC) comparison].

#### 3.3.1 Model 1

First, we created a linear regression model to assess the relationships between functional connectivity and spatial learning using the MWM training data (Day 1–Day 4 latency to escape). The model established the weights (coefficients) of a linear combination of functional connections that predicts spatial learning in the MWM training data. This model relates the rsFC changes (APP/PS1 compared to WT) to the spatial learning performance in each individual mouse. The coefficients determined by the model relate to the strength of the predictive power of each connection. A positive coefficient means that as the strength of that connection increased, there was an increase in learning as defined by difference in latency to escape over the course of the training phase (Table 6).

**Table 6:** Model 1_Relationship between spatial learning and functional connectivity.

**6-month**		
**Region 1**	**Region 2**	**Coefficient**
Temporal	Hippocampus	26.82420986
Orbital	Prelimbic	−24.09031887
Temporal	Ectorhinal	19.16297608
Temporal	Perirhinal	−12.76793136
Insula	Striatum	12.05063559
Dentate Gyrus	Thalamus	−10.16480068
Anterior Cingulate	Perirhinal	9.797571525
Anterior cingulate	Striatum	−9.797490263
Perirhinal	Midbrain	−9.784945678
Piriform	Cortical subplate	−8.806284779
Ectorhinal	Hippocampus	−8.610174302
Entorhinal	Hippocampus	−7.811033297
Cortical subplate	Ectorhinal	−6.884725909
Prelimbic	Anterior cingulate	5.846948876
Piriform	Entorhinal	5.567930665
Insula	Piriform	−5.434191692
Thalamus	Striatum	−5.147431246
Cortical subplate	Striatum	−4.624752929
Hippocampus	Dentate gyrus	−3.547783071
Subiculum	Midbrain	2.52006327
Anterior cingulate	Ectorhinal	−2.370938573
Cortical subplate	Entorhinal	2.319090821
Ectorhinal	Perirhinal	−2.273131213
Cortical subplate	Perirhinal	2.23796394
Dentate gyrus	Subiculum	2.187598044
Entorhinal	Dentate gyrus	1.751455894
Perirhinal	Hippocampus	−1.671078314
Midbrain	Thalamus	1.279774685
Temporal	Striatum	0.384806112
Ectorhinal	Entorhinal	0.321562375
Insula	Dentate gyrus	−0.244430174
Perirhinal	Entorhinal	−0.224729829
Cortical subplate	Hippocampus	−0.206707249
Insula	Cortical subplate	0.163654833
Hippocampus	Thalamus	0.054958822
Orbital	Anterior cingulate	−0.034507619
Entorhinal	Subiculum	0.000320998
**10-month**		
**Region 1**	**Region 2**	**Coefficient**
Insula	Ectorhinal	21.47025733
Subiculum	Dentate gyrus	15.55299204
Orbital	Hypothalamus	−9.313354789
Midbrain	Retrosplenial	8.467112143
Dentate gyrus	Hippocampus	−7.059569616
Anterior cingulate	Striatum	−6.135849639
Prelimbic	Striatum	−6.045696026
Cortical subplate	Hypothalamus	−5.601318258
Striatum	Temporal	−4.976794654
Thalamus	Midbrain	−4.001974207
Entorhinal	Hippocampus	2.56408521
Entorhinal	Dentate gyrus	1.030937158
Midbrain	Subiculum	0.475612025
Piriform	Entorhinal	0.368271799
Ectorhinal	Hippocampus	0.308919583
Insula	Striatum	0.252494772
Entorhinal	Subiculum	0.033419686
Prelimbic	Cortical subplate	0.000366419

Functional connections that have explanatory power for spatial learning performance in 6- and 10-month APP/PS1 mice. Each row depicts a functional connection between two brain regions. Order of regions (Region 1 or Region 2) does not imply directionality of the connection. Only connections that the model identified as significant are shown. The coefficient is a measure of predictive power for behavioral performance.

In the 6-month cohort, the model found 37 total connections that have predictive value for spatial learning performance (R > 0.99). 17 connections were shown to have a positive coefficient, indicating a positive relationship with learning performance. 20 connections were shown to have a negative coefficient, indicating a negative relationship with learning performance (Figure 4C). In the 10-month cohort, 18 connections were found to be predictive of learning performance (R > 0.99). 11 connections were shown to have a positive coefficient, indicating a positive relationship with learning performance. 7 connections were shown to have a negative coefficient, indicating a negative relationship with learning performance (Figure 4D). Across time points, both models found a positive coefficient for connections including the subiculum and dentate gyrus, subiculum and entorhinal, piriform and entorhinal, insula and striatum. A negative correlation was found for connections including the dentate gyrus and hippocampus, striatum and anterior cingulate. The model also found connections that changed directionality; the striatum and temporal as well as the thalamus and midbrain had a positive coefficient at the 6-month time point but a negative coefficient at the 10-month time point; the hippocampus and entorhinal had a negative coefficient at the 6-month time point and a positive coefficient at the 10-month time point.

#### 3.3.2 Model 2

Model 2 established the relationships between functional connectivity and spatial memory using the MWM probe trial data (% time spent in SE quadrant). In this model, a positive coefficient means that as the strength of that connection increased, there was an increase in memory as defined by time spent in the SE quadrant (Table 7).

**Table 7:** Model 2_Relationship between spatial memory and functional connectivity.

**6-months**		
**Region 1**	**Region 2**	**Coefficient**
Dentate gyrus	Subiculum	24.91927776
Orbital cortex	Prelimbic	−16.79821157
Entorhinal	Hippocampus	−8.555816499
Anterior cingulate	Striatum	−7.667657046
Piriform	Entorhinal	−5.438188566
Ectorhinal	Entorhinal	4.485144333
Hippocampus	Dentate Gyrus	−4.022499433
Midbrain	Thalamus	−3.865921728
Cortical Subplate	Perirhinal	−1.547348309
**10-months**		
**Region 1**	**Region 2**	**Coefficient**
Cortical subplate	Entorhinal	17.6031419
Anterior cingulate	Hippocampus	16.9836591
Anterior cingulate	Prelimbic	14.48779041
Prelimbic	Cortical subplate	10.52685047
Orbital	Hypothalamus	−9.706540747
Ectorhinal	Perirhinal	8.323748089
Thalamus	Midbrain	−6.584637362
Anterior cingulate	Retrosplenial	−5.100520463
Perirhinal	Subiculum	−4.559392347
Cortical subplate	Hippocampus	−4.159240508
Insula	Cortical subplate	3.083394225
Insula	Ectorhinal	2.165915123
Ectorhinal	Dentate gyrus	−1.959011074
Anterior cingulate	Insula	1.120018192
Orbital	Prelimbic	−0.735121997
Prelimbic	Striatum	−0.006172407
Anterior cingulate	Orbital	−5.97E−05

Functional connections that have explanatory power for spatial memory performance in 6- and 10-month APP/PS1 mice. Each row depicts a functional connection between two brain regions. Order of regions (Region 1 or Region 2) does not imply directionality of the connection. Only connections that the model identified as significant are shown. The coefficient is a measure of predictive power for behavioral performance.

In the 6-month cohort, the model found nine total connections that have predictive value for spatial memory performance (R > 0.99). 2 connections were shown to have a positive coefficient, indicating a positive relationship with memory performance. 7 connections were shown to have a negative coefficient, indicating a negative relationship with memory performance (Figure 4E). In the 10-month cohort, 17 connections were found to be predictive of memory performance (R > 0.99). 11 connections were shown to have a positive coefficient, indicating a positive relationship with memory performance. 6 connections were shown to have a negative coefficient, indicating a negative relationship with memory performance (Figure 4F). The model identified one connection that has a negative coefficient across both time points: thalamus and midbrain. The model identified one connection that changed directionality between time points: the prelimbic cortex and orbital cortex had a negative coefficient at the 6-month time point and a positive coefficient at the 10-month time point.

### 3.4 Assessing model performance

To assess model performance, we performed 10,000 bootstrap runs to identify the upper and lower bounds of the confidence interval of the coefficient for each identified connection (Supplementary Tables 6, 7). On 50–70% of runs for each model, the sign of each coefficient (positive or negative) remained consistent. On ~35–40% of runs, the coefficient was zero, which is consistent with the LASSO regularization approach. The exception is the Model 1: 6 month, where coefficients were zero on only 12% of runs.

## 4 Discussion

This study elucidated the changes in FC and investigates the relationship between FC and cognitive decline in an Aβ mouse model of AD across disease progression. Utilizing rs-fMRI in awake, unanesthetized mice, we observed significant alterations in FC across all three time points (3-, 6-, and 10-months). FC changes at 6- and 10-months were shown to have explanatory power for spatial learning and memory.

### 4.1 Morris Water Maze

The MWM assay revealed that APP/PS1 mice exhibit significant deficits in spatial learning and memory by 10 months of age, with some of evidence of early spatial memory deficits in 6-month APP/PS1 mice. The absence of significant differences at 3-months of age underscores the importance of examining early connectivity changes that precede overt cognitive decline.

### 4.2 Functional connectivity alterations

Our results indicate a trend of progressive hyperconnectivity in APP/PS1 mice across all observed time points, with 47 regions exhibiting hyperconnectivity at 3-months and 84 at 10-months. This progressive increase in hyperconnectivity suggests a continuous alteration in brain network dynamics as the disease advances. At 3 months of age, these mice do not exhibit learning and memory deficits but show a clear pattern of hyperconnectivity. At this age point, these mice do not have Aβ plaques but have been shown to have an increase in levels of soluble Aβ (Zhou et al., 2021). The hyperconnectivity at the age point may compensate for soluble Aβ− induced changes in neural signaling (Ben-Nejma et al., 2019). However, as the disease progresses, the significant increase in hyperconnectivity may contribute to network dysfunction as in the 10-month time point. Alternatively, this increase in hyperconnectivity could represent a failed compensatory mechanism to preserve cognitive function.

Recent human PET imaging studies have proposed a link between the accumulation of Aβ plaques and functional hyperconnectivity in the brain (Schultz et al., 2017; Sepulcre et al., 2017; Sintini et al., 2021; Wales and Leung, 2021). Our data may support this hypothesis and extend this finding to early disease stages prior to plaque deposition. At 3-months, soluble Aβ may be primarily contributing to the hyperconnectivity we see at this disease stage. At 6- and 10-months, both soluble and insoluble forms of Aβ may contribute to the hyperconnectivity. The significant increase of Aβ plaque load at 10-months supports the observed increase in the number of hyperconnected regions. Future studies are necessary to understand the contribution of soluble and insoluble forms of Aβ.

Several studies have documented hyperconnectivity in mouse models of AD. For instance, Kesler et al. (2018) investigated the 5XFAD transgenic mouse model and observed hyperconnectivity in memory-related networks at early disease stages, particularly in mice aged 3–6 months (Kesler et al., 2018). Bero et al. (2012) reported hyperconnectivity in the DMN using the PDAPP mouse model at 6 months of age, highlighting increased connectivity as a potential early indicator of AD pathology (Bero et al., 2012). Other studies have observed early hyperconnectivity followed by a decrease in functional connectivity as the disease progresses. For example, Morrissey et al. (2022) observed higher interhemispheric connectivity between hippocampal subregions, followed by a later decrease in this measure in an APP knock-on model (*App*^*NL*−*G*−*F*/*NL*−*G*−F^) (Morrissey et al., 2022). In contrast, other studies have reported hypoconnectivity at early-stages of disease progression. In the 3xTG-AD mouse mode, Manno et al. (2019) found decreased interhemispheric hippocampal functional at early stages of disease progression, and a study conducted Mandino et al. (2022) found a deficit in regional homogeneity (a measure of localized functional connectivity) in memory related regions including the amygdala, striatum, prefrontal cortex, and hippocampus in early-mid stages of disease progression in 3xTG-AD mice (Mandino et al., 2022).

The differences in results between studies on functional connectivity in AD mouse models can largely be attributed to variations in the mouse models used. Different mouse models of AD, such as 5XFAD, APP/PS1, and 3xTg-AD, exhibit distinct pathological features and progression rates, which can lead to different patterns of functional connectivity across disease progression. Analysis methods also play a critical role; discrepancies can arise from using different imaging techniques, data processing pipelines, and statistical methods for measuring and interpreting functional connectivity. Hasani et al. (2021) have discussed the conflicting findings of FC studies in depth. Of note, analyses of global connectivity (including the ROI-to-ROI methodology used here) are more likely to report overall hyperconnectivity compared to analyses of local network activity.

Additionally, an important aspect of our studies is that all imaging is conducted in awake, unanesthetized mice. The use of anesthetics has been shown to significantly alter functional imaging outputs, including FC (Grandjean et al., 2014; Jonckers et al., 2014; Paasonen et al., 2018; Fadel et al., 2022). We use an acclimation protocol to reduce animal stress, which was previously verified with measurements of cortisol between naïve, un-acclimated, and acclimated mice (Fadel et al., 2022). Our data demonstrated that at the end of the acclimation period, the cortisol levels of mice had returned to pre acclimation levels. Given the variation in results across preclinical studies, removing the potentially confounding effects of anesthesia provides increased translational relevance.

### 4.3 Default mode network alterations

Our analysis of DMN-like regions (N. Xu et al., 2022) revealed early hyperconnectivity at 3-months. However, unlike global changes, we saw a decrease in hyperconnected DMN regions at 6 and 10-months. These results could indicate a transition from compensatory hyperactivity to network breakdown and hypoconnectivity in later stages of disease. Despite the reduction in hyperconnected regions in the DMN at later stages, certain connections, such as those between the hippocampus and dentate gyrus, remained persistently hyperconnected across all time points. This persistent hyperconnectivity is significant because the hippocampus and dentate gyrus are crucial for memory processes, which are profoundly affected in AD (Bonanni et al., 2021; Schultz et al., 2017; Stoub et al., 2006).

Schultz et al. (2017) found DMN hyperconnectivity in amyloid positive individuals, while DMN hypoconnectivity was seen in individuals who were both amyloid and tau positive, suggesting that the shift to hypoconnectivity may be mediated by tau. Additionally, Hampton et al. (2020) found that decreased activity in the DMN predicted neurodegeneration in DMN regions. A review by Wales and Leung (2021) noted amyloid-related hyper-connectivity and tau-related hypo-connectivity, and proposed that conflicting reports may reflect varying contributions of amyloid and tau. In our study, we use an amyloid beta mouse model that does not exhibit overt tau pathology or neurodegeneration at the time points used for this study and therefore may not experience this shift to decreased FC.

### 4.4 Machine learning models

For the first time, we used machine learning to establish significant relationships between FC changes and spatial learning (Model 1) and memory performance (Model 2). The models identified specific ROI-to-ROI connections that were predictive of spatial learning and memory performance, providing novel insights into how FC alterations can serve as early indicators of cognitive decline. The identification of both positive and negative coefficients for various connections indicates that increased FC predicts both positive and negative effects on behavior performance, reflecting the complex nature of AD-related network changes. Positive coefficients may represent compensatory strategies for memory performance, while negative coefficients result when aberrant activity disrupts neuronal processing.

The machine learning models revealed several key connections that are predictive of cognitive performance. Model 1 identified temporal-hippocampus as the strongest predictor of learning in the 6-month cohort, with a positive coefficient indicating learning performance improves as the strength of this connection increases. The second strongest predictor at this time point was the orbital-prelimbic connection, with a negative coefficient indicating learning performance decreases as the strength of this connection increases. In the 10-month cohort, Model 1 identified insula-ectorhinal cortex and subiculum-dentate gyrus as the strongest predictors of learning performance. Temporal cortex connections are highly predictive of learning performance at the 6-month time point (temporal-hippocampus, temporal-ectorhinal, and temporal-perirhinal are in the top five predictors), but not at 10-months (only temporal-striatum was identified), suggesting a shift in the networks engaged for learning. These results suggest that as the disease progresses, there is a loss of engagement of the temporal association areas, and a corresponding decrease in learning performance. The shift in regions identified by the Model may reflect underlying deterioration of normal cognitive processes.

Model 2 identified dentate gyrus-subiculum (positive coefficient) as the strongest predictor of memory performance in the 6-month cohort. The second strongest predictor at this time point was the orbital-prelimbic connection (negative coefficient), and 7/9 connections identified had negative coefficients. Memory deficits begin to emerge at the 6-month time point, and the negative coefficients identified by the Model may indicate that aberrant hyperconnectivity is disruptive to memory performance. In the 10-month cohort, Model 2 identified the cortical sublate-entorhinal and anterior cingulate-hippocampus as the strongest predictors of memory performance (positive coefficients). At this time point, the APP/PS1 mice have very low spatial memory performance, and these changes identified by the model are not able to compensate as pathology accumulates. The differences in these two models likely reflect differences in underlying cognitive processes between learning and memory.

Our modeling choices were parsimonious: we adopted linear models and careful statistical approaches (initial variable selection, LASSO regularization, and leave-one-out cross validation) to ensure rigorous models despite relatively small sample sizes. LASSO regularization optimizes model performance and performs variable selection by discarding non-contributory variables. In bootstrap analyses, the confidence intervals often include zero for coefficients optimized by LASSO. Our results indicate that in 50–70% of the bootstrap runs (on average), the sign of the coefficient was consistent with the original model. In the remaining runs, approximately 40% of the bootstrap samples resulted in the coefficient being exactly zero. Only for Model 1: 6-month data, this fraction was 12%. This ensures robust interpretations regarding the roles of the variables. The models revealed valuable insights, but we anticipate that non-linear models that allow for variable-interactions could explain more variations in the data. Replicating the modeling in additional mouse models of AD and extending the use of the modeling to human data would provide additional insight into the progression of AD.

### 4.5 Implications for AD diagnosis and treatment

The early detection of FC changes, especially hyperconnectivity in memory-related networks, could serve as a biomarker for preclinical AD, allowing for timely therapeutic interventions. Furthermore, the observed decrease in number of hyperconnected regions in the DMN-like regions provides valuable insights into the temporal dynamics of network changes in AD. The initial phase of hyperconnectivity might represent a window of opportunity for therapeutic interventions aimed at enhancing network resilience. Targeting the persistent hyperconnectivity in memory-related regions could help to preserve cognitive functions and slow the progression of the disease.

The identified connections from the machine learning model could also be pivotal in providing targets for potential therapeutic strategies aimed at modulating network activity to mitigate cognitive decline in human patients. For example, therapies designed to enhance connectivity in pathways with positive coefficients could help reinforce networks that support memory and learning. On the other hand, interventions that target pathways with negative coefficients could aim to reduce pathological hyperconnectivity, thus restoring normal network function.

### 4.6 Conclusions

For the first time, we established the relationship between behavior and functional connectivity changes in awake, unanesthetized APP/PS1 mice compared to WT controls. This study demonstrated that significant changes in FC precede cognitive deficits in an Aβ mouse model of AD. The observed trend of progressive hyperconnectivity in memory-related regions could initially represent a compensatory response to maintain cognitive functions. However, as the disease progresses, this hyperactivity expands to involve more regions, contributing to network dysfunction. Interestingly, we saw a loss hyperconnectivity in DMN-like regions, underscoring the dynamic nature of neural network alterations in AD. The use of rs-fMRI to detect these changes highlights its potential as a valuable tool for early detection of AD. Furthermore, the novel use of machine learning methods is likely to provide a framework for consistent early detection of AD.

## Data Availability

The raw data supporting the conclusions of this article will be made available by the authors, without undue reservation.
