# Supplementary material for: Modeling functional connectivity with learning and memory in a mouse model of Alzheimer's disease
Source: Front Neuroimaging. 2025 Apr 25;4:1558759. doi: 10.3389/fnimg.2025.1558759 (PMC12062036; doi:10.3389/fnimg.2025.1558759)
Supplement: Supplementary file 1 [file Table_1.docx]

## Supplementary Material


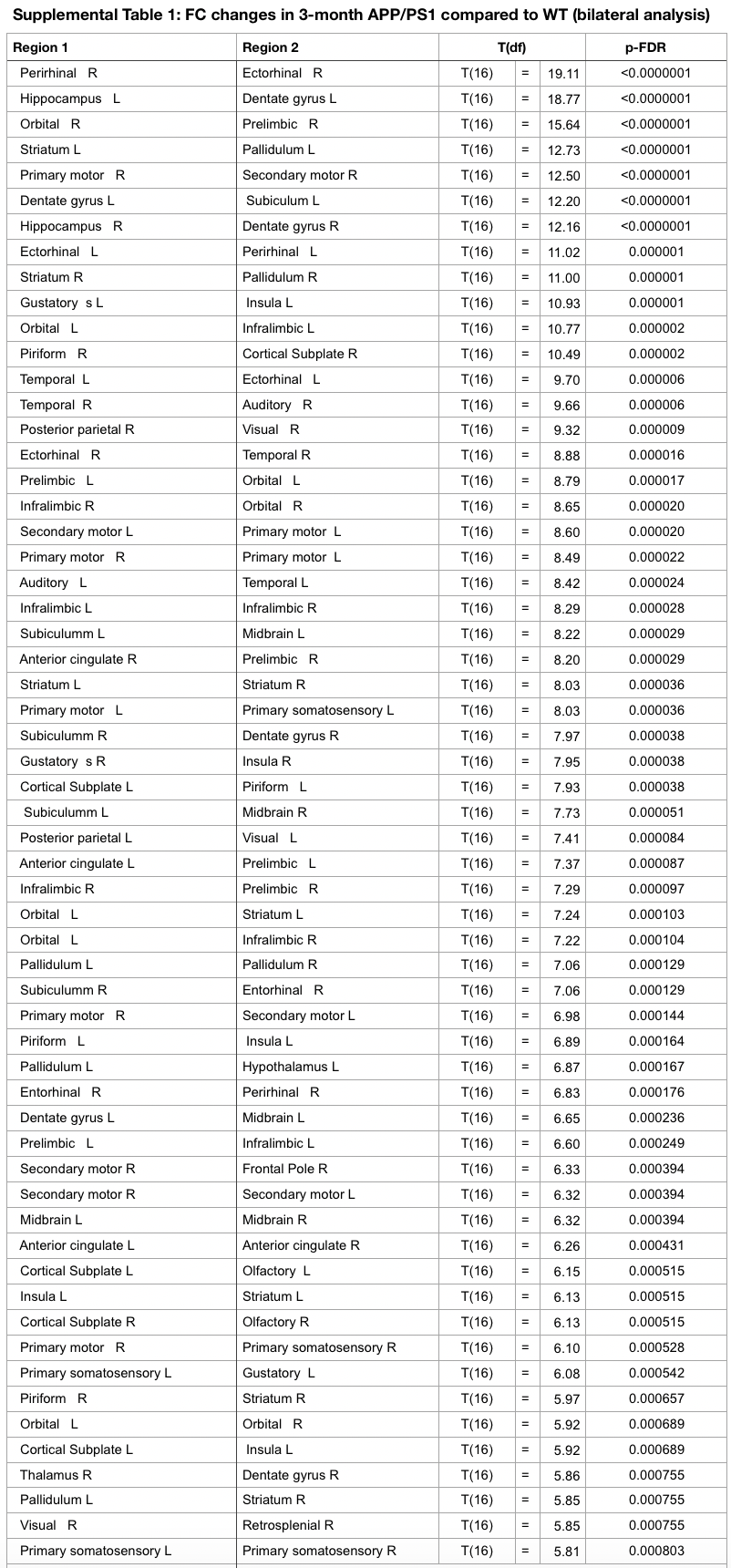


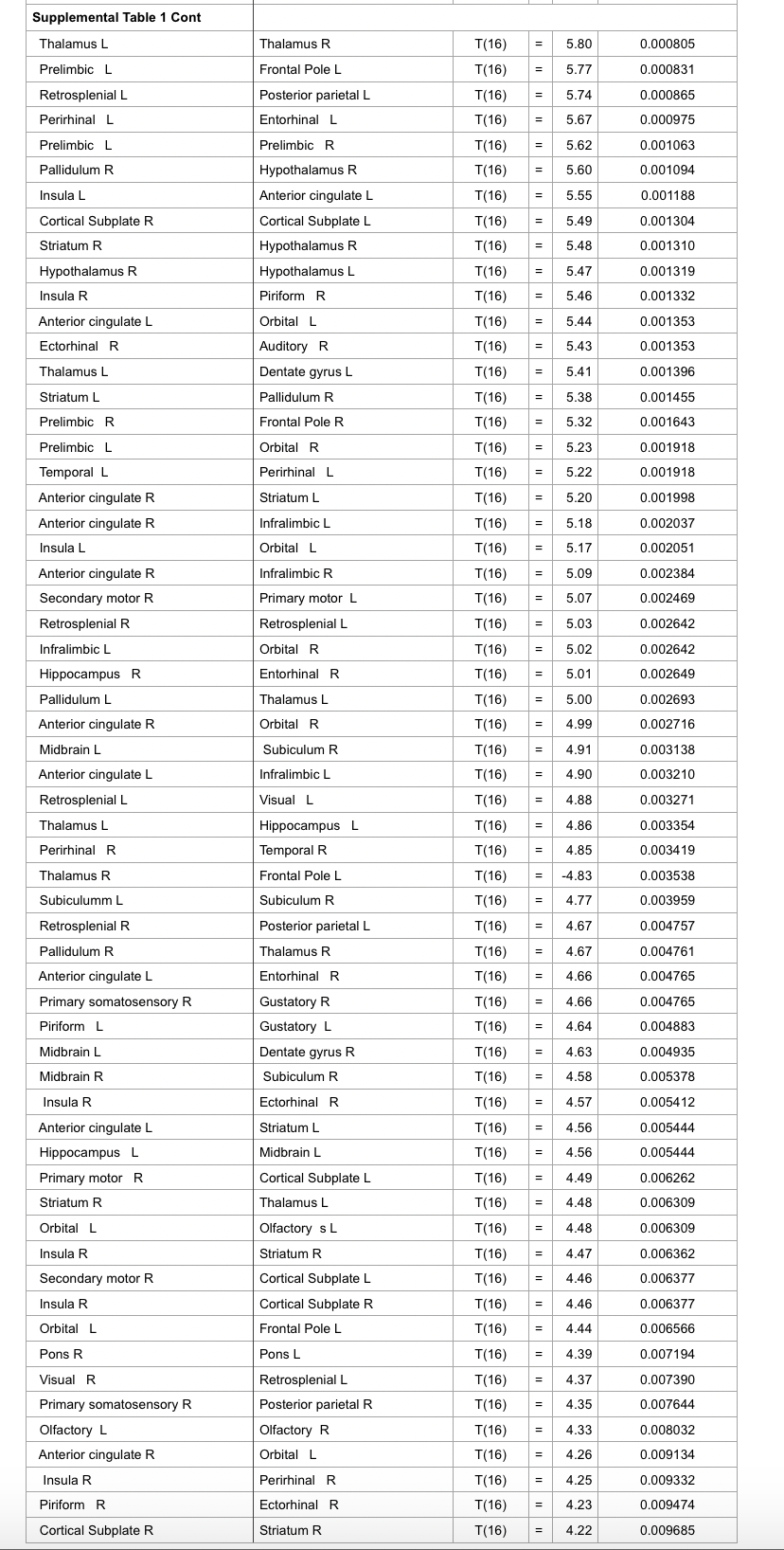


Supplemental Table 1: 3-month functional connectivity changes in APP/PS1 compared to WT, bilateral analysis (Left and Right hemispheres). Each row depicts a functional connection between two brain regions. Order of regions (Region 1 or Region 2) does not imply directionality of the connection. Only connections that have significant differences between APP/PS1 and WT mice are shown. All comparisons based on a linear regression model, APP/PS1 > WT. Both uncorrected (p unc) and FDR-corrected (p FDR) p-values are given.


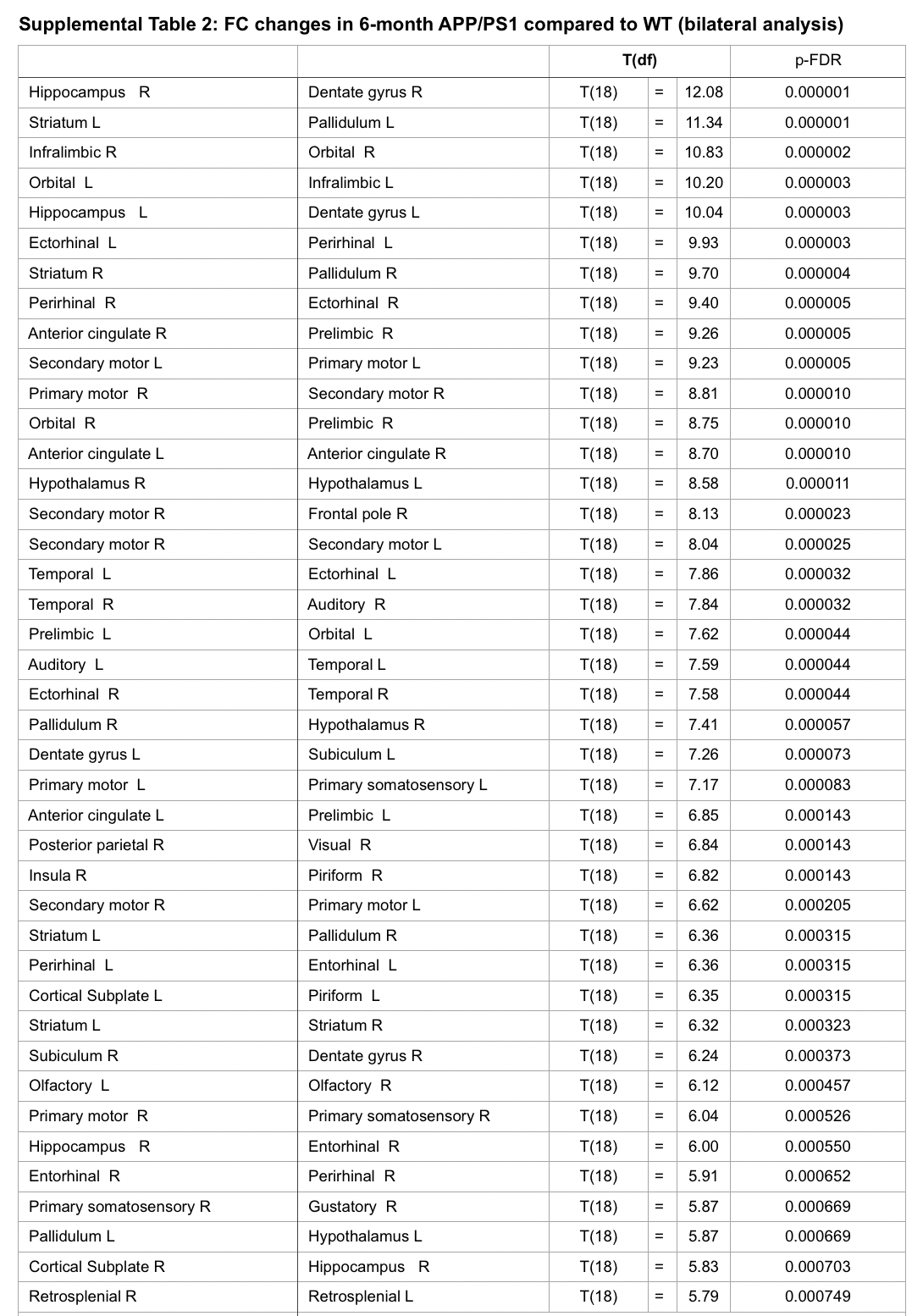


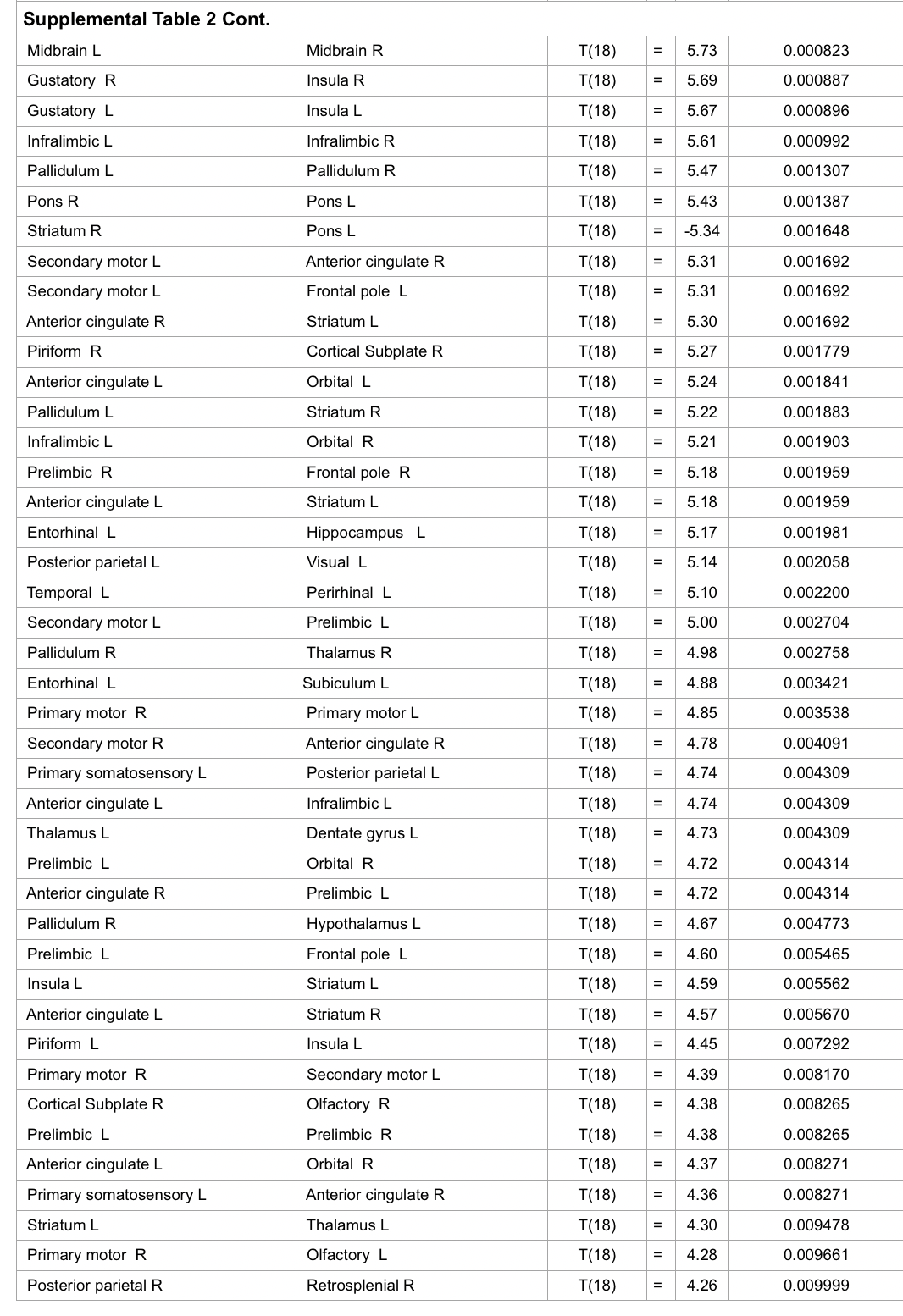
 Supplemental Table 2: 6-month functional connectivity changes in APP/PS1 compared to WT, bilateral analysis (Left and Right hemispheres). Each row depicts a functional connection between two brain regions. Order of regions (Region 1 or Region 2) does not imply directionality of the connection. Only connections that have significant differences between APP/PS1 and WT mice are shown. All comparisons based on a linear regression model, APP/PS1 > WT. Both uncorrected (p unc) and FDR-corrected (p FDR) p-values are given.


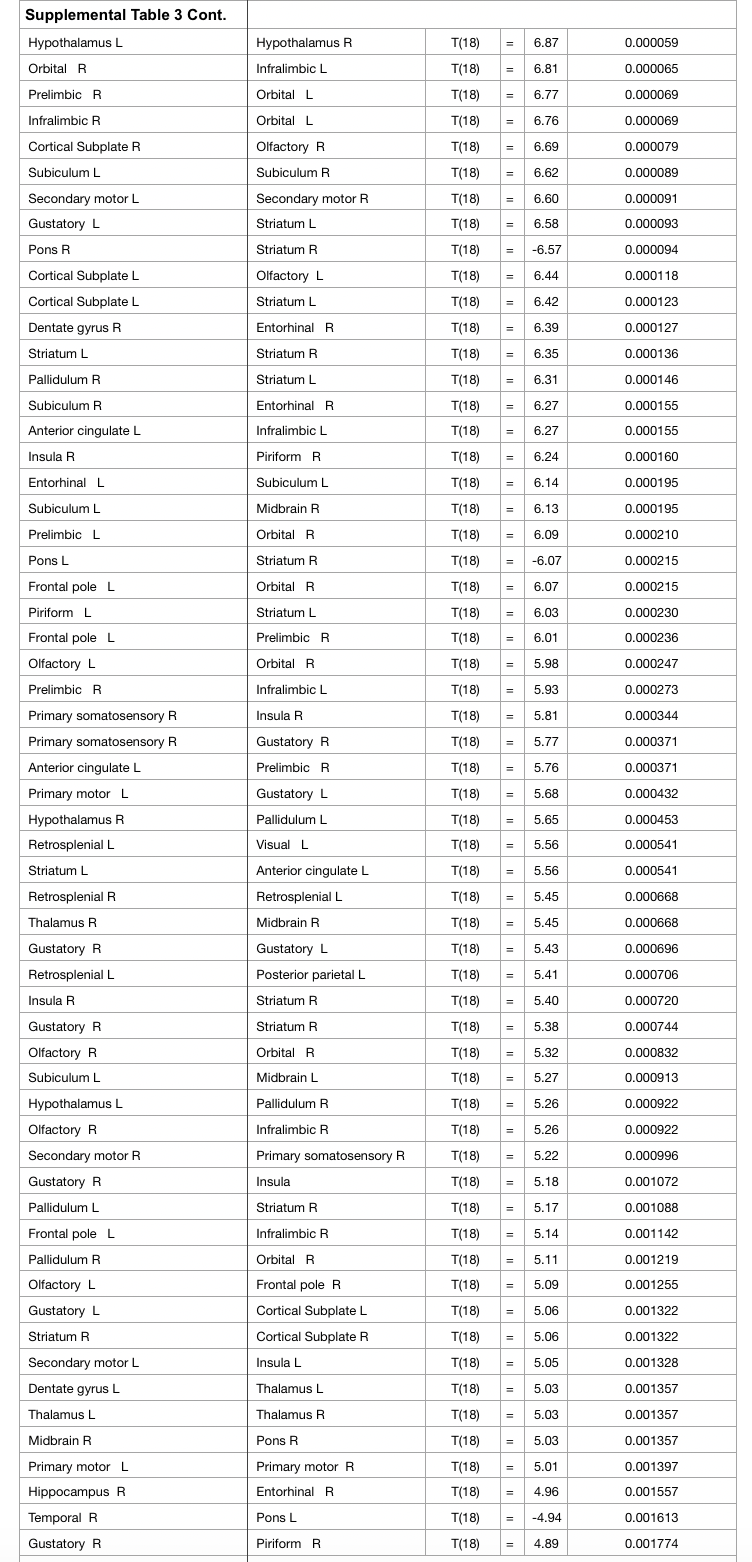

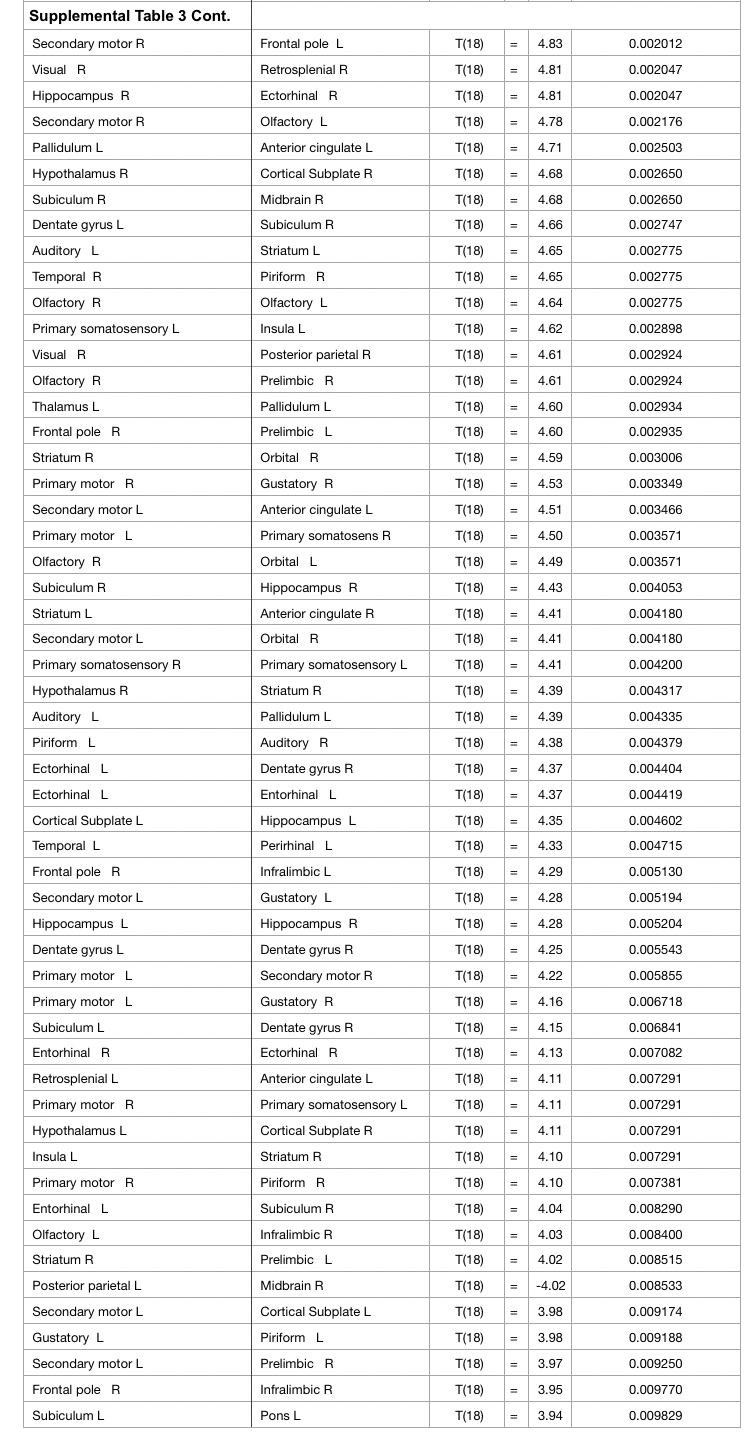

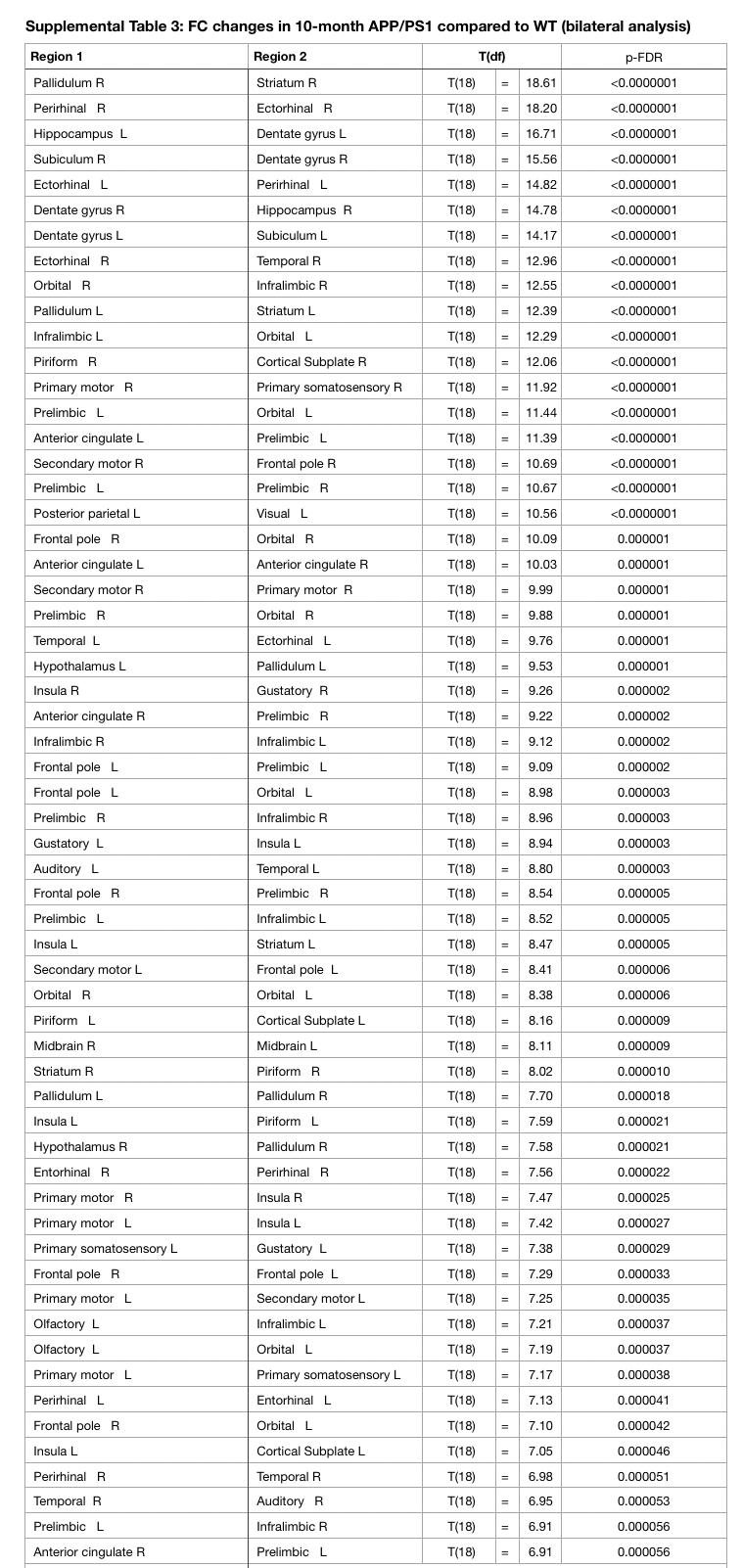


Supplemental Table 3: 10-month functional connectivity changes in APP/PS1 compared to WT, bilateral analysis (Left and Right hemispheres). Each row depicts a functional connection between two brain regions. Order of regions (Region 1 or Region 2) does not imply directionality of the connection. Only connections that have significant differences between APP/PS1 and WT mice are shown. All comparisons based on a linear regression model, APP/PS1 > WT. Both uncorrected (p unc) and FDR-corrected (p FDR) p-values are given.

|  |  | p-value | p-FDR |
| --- | --- | --- | --- |
| Hippocampus_Field_CA1 | Pons | 3.33E-22 | 0.000108 |
| Striatum | Pallidulum | 1.95E-15 | 0.000215 |
| Dentate_gyrus | Pons | 4.18E-14 | 0.000323 |
| Hippocampus_Field_CA1 | Midbrain | 9.74E-14 | 0.00043 |
| Entorhinal_area | Cortical_Subplate | 1.15E-13 | 0.000538 |
| Perirhinal_area | Dentate_gyrus | 4.77E-12 | 0.000645 |
| Piriform_area | Para-_Post-subiculum | 1.51E-09 | 0.000753 |
| Piriform_area | Cortical_Subplate | 2.76E-09 | 0.00086 |
| Cortical_Subplate | Pallidulum | 3.3E-09 | 0.000968 |
| Perirhinal_area | Entorhinal_area | 9.35E-09 | 0.001075 |
| Hippocampus_Field_CA1 | Dentate_gyrus | 8.8E-08 | 0.001183 |
| Pallidulum | Hypothalamus | 1.32E-07 | 0.00129 |
| Agranular_insular_area | Entorhinal_area | 3.41E-07 | 0.001398 |
| Perirhinal_area | Pons | 3.77E-07 | 0.001505 |
| Pallidulum | Thalamus | 5.96E-07 | 0.001613 |
| Thalamus | Midbrain | 8.36E-07 | 0.00172 |
| Hippocampus_Field_CA1 | Entorhinal_area | 9.17E-07 | 0.001828 |
| Thalamus | Pons | 1.22E-06 | 0.001935 |
| Ectorhinal_area | Pons | 1.58E-06 | 0.002043 |
| Pallidulum | Midbrain | 1.72E-06 | 0.002151 |
| Dentate_gyrus | Para-_Post-subiculum | 2.74E-06 | 0.002258 |
| Dentate_gyrus | Thalamus | 7.98E-06 | 0.002366 |
| Agranular_insular_area | Striatum | 1.2E-05 | 0.002473 |
| Visceral_area | Pons | 1.86E-05 | 0.002581 |
| Orbital_area | Entorhinal_area | 3.14E-05 | 0.002688 |
| Striatum | Pons | 4.22E-05 | 0.002796 |
| Anterior_cingulate_area | Agranular_insular_area | 4.65E-05 | 0.002903 |
| Gustatory_areas | Entorhinal_area | 5.42E-05 | 0.003011 |
| Hypothalamus | Midbrain | 6.63E-05 | 0.003118 |
| Posterior_parietal_association_areas | Para-_Post-subiculum | 8.92E-05 | 0.003226 |
| Ectorhinal_area | Dentate_gyrus | 0.000106 | 0.003333 |
| Ectorhinal_area | Midbrain | 0.000173 | 0.003441 |
| Olfactory_areas | Para-_Post-subiculum | 0.000191 | 0.003548 |
| Agranular_insular_area | Cortical_Subplate | 0.00021 | 0.003656 |
| Piriform_area | Striatum | 0.000233 | 0.003763 |
| Auditory_area | Olfactory_areas | 0.000408 | 0.003871 |
| Piriform_area | Entorhinal_area | 0.000421 | 0.003978 |
| Visceral_area | Cortical_Subplate | 0.000459 | 0.004086 |
| Visual_area | Para-_Post-subiculum | 0.000477 | 0.004194 |
| Secondary_motor_area | Cortical_Subplate | 0.000506 | 0.004301 |
| Primary_somatosensory_area | Pons | 0.000616 | 0.004409 |
| Visceral_area | Entorhinal_area | 0.000644 | 0.004516 |
| Primary_somatosensory_area | Striatum | 0.000656 | 0.004624 |
| Ectorhinal_area | Entorhinal_area | 0.00087 | 0.004731 |
| Auditory_area | Hypothalamus | 0.00089 | 0.004839 |
| Temporal_association_areas | Midbrain | 0.000914 | 0.004946 |
| Agranular_insular_area | Secondary_motor_area | 0.000948 | 0.005054 |
| Orbital_area | Striatum | 0.001232 | 0.005161 |
| Hippocampus_Field_CA1 | Cortical_Subplate | 0.001594 | 0.005269 |
| Para-_Post-subiculum | Striatum | 0.001702 | 0.005376 |
| Posterior_parietal_association_areas | Retrosplenial_area | 0.001847 | 0.005484 |
| Primary_somatosensory_area | Midbrain | 0.001982 | 0.005591 |
| Agranular_insular_area | Pallidulum | 0.002015 | 0.005699 |
| Temporal_association_areas | Auditory_area | 0.002251 | 0.005806 |
| Visceral_area | Gustatory_areas | 0.002745 | 0.005914 |
| Entorhinal_area | Midbrain | 0.002874 | 0.006022 |
| Agranular_insular_area | Primary_motor_area | 0.002902 | 0.006129 |
| Olfactory_areas | Cortical_Subplate | 0.002913 | 0.006237 |
| Anterior_cingulate_area | Visceral_area | 0.003259 | 0.006344 |
| Gustatory_areas | Pons | 0.003405 | 0.006452 |
| Primary_motor_area | Piriform_area | 0.003752 | 0.006559 |
| Hippocampus_Field_CA1 | Striatum | 0.00385 | 0.006667 |
| Ectorhinal_area | Cortical_Subplate | 0.003934 | 0.006774 |
| Para-_Post-subiculum | Thalamus | 0.004187 | 0.006882 |
| Anterior_cingulate_area | Pallidulum | 0.004317 | 0.006989 |
| Visual_area | Dentate_gyrus | 0.004878 | 0.007097 |
| Agranular_insular_area | Pons | 0.005472 | 0.007204 |
| Para-_Post-subiculum | Midbrain | 0.005779 | 0.007312 |
| Primary_motor_area | Striatum | 0.005961 | 0.007419 |
| Ectorhinal_area | Perirhinal_area | 0.005982 | 0.007527 |
| Temporal_association_areas | Hypothalamus | 0.006267 | 0.007634 |
| Cortical_Subplate | Pons | 0.007034 | 0.007742 |

Supplemental Table 4: Results of 2-way ANOVA analysis of all three age cohorts of APP/PS1 and WT mice. The results specifically show the p-values and corresponding p-FDR for effects of mouse age for each connection between brain regions. Only significant results are shown.

| Model name | AIC using all significant connections | AIC using only the DMN/memory connections |
| --- | --- | --- |
| Model-1 6-month | 51.7027 | -57.5586 |
| Model-1 10-month | 12.5374 | -73.2138 |
| Model-2 6-month | 45.5731 | -30.1227 |
| Model-2 10-month | 60.9485 | -75.7313 |

Supplemental Table 5: Akaike information criterion (AIC) values for each model using all significantly altered connections compared to filtering connections for DMN and memory-related regions. For each model, filtering to the biologically relevant connections decreased AIC values. A lower AIC value gives more empirical support for the model (Cavanaugh and Neath, 2019).

 Supplemental Table 6: Modeling the relationship between learning and FC. Coefficient represents the strength of the predictive power for the connection. Lower and upper bounds of the coefficient identified using 10,000 bootstrap runs. Positive, negative, and zero fraction represent the proportion of runs on which the model identified the coefficient as positive, negative, or zero, respectively.


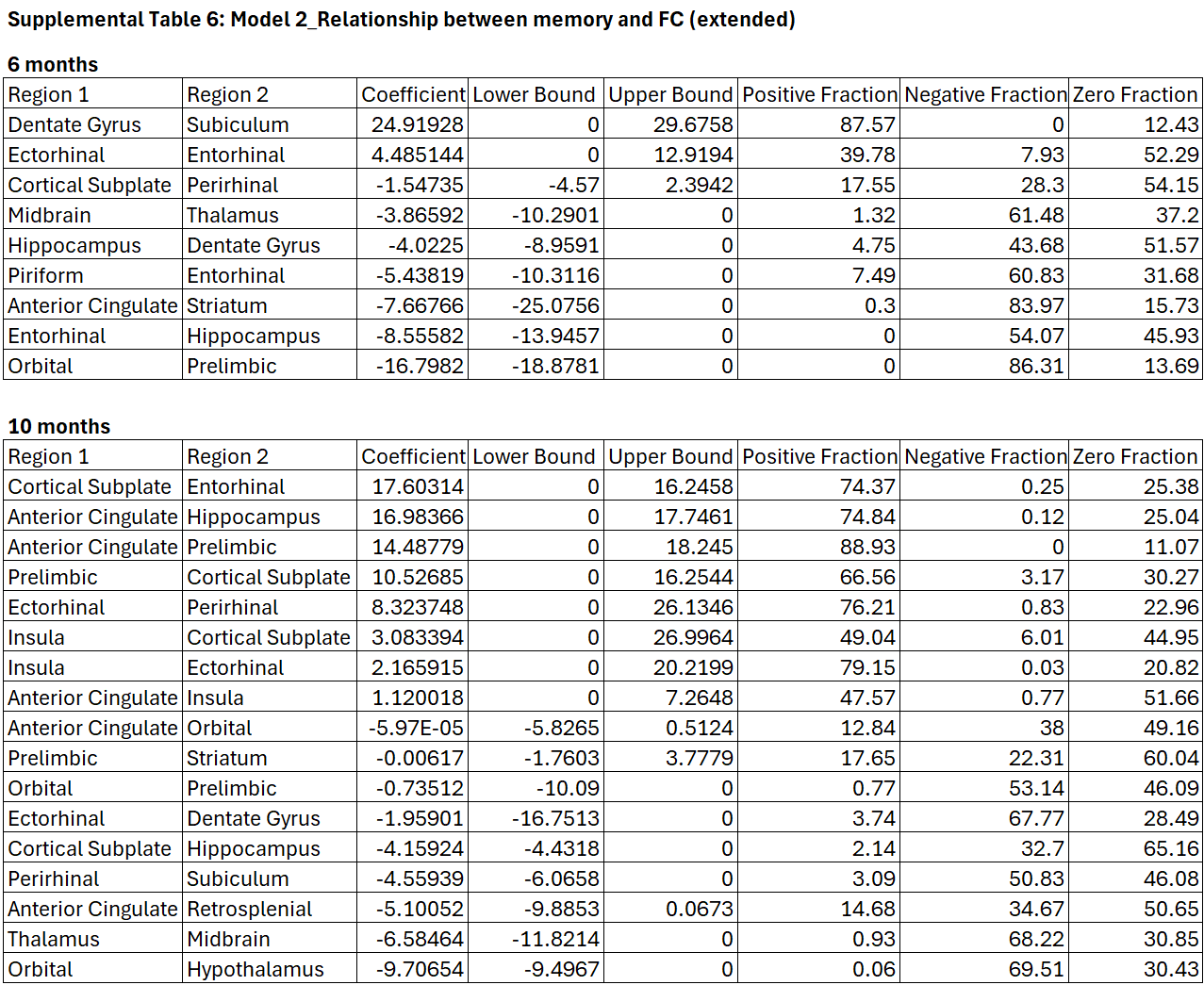


Supplemental Table 7: Modeling the relationship between memory and FC. Coefficient represents the strength of the predictive power for the connection. Lower and upper bounds of the coefficient identified using 10,000 bootstrap runs. Positive, negative, and zero fraction represent the proportion of runs on which the model identified the coefficient as positive, negative, or zero, respectively.
